# Physical activity levels in adults and older adults 3–4 years after pedometer-based walking interventions: Long-term follow-up of participants from two randomised controlled trials in UK primary care

**DOI:** 10.1371/journal.pmed.1002526

**Published:** 2018-03-09

**Authors:** Tess Harris, Sally M. Kerry, Elizabeth S. Limb, Cheryl Furness, Charlotte Wahlich, Christina R. Victor, Steve Iliffe, Peter H. Whincup, Michael Ussher, Ulf Ekelund, Julia Fox-Rushby, Judith Ibison, Stephen DeWilde, Cathy McKay, Derek G. Cook

**Affiliations:** 1 Population Health Research Institute, St George’s University of London, London, United Kingdom; 2 Pragmatic Clinical Trials Unit, Queen Mary’s University of London, London, United Kingdom; 3 Gerontology and Health Services Research Unit, Brunel University, London, United Kingdom; 4 Research Department of Primary Care & Population Health, University College London, London, United Kingdom; 5 Department of Sport Medicine, Norwegian School of Sport Sciences, Oslo, Norway; 6 MRC Epidemiology Unit, University of Cambridge, Cambridge, United Kingdom; 7 Health Economics Research Group, Brunel, University of London, London, United Kingdom; Stanford University, UNITED STATES

## Abstract

**Background:**

Physical inactivity is an important cause of noncommunicable diseases. Interventions can increase short-term physical activity (PA), but health benefits require maintenance. Few interventions have evaluated PA objectively beyond 12 months. We followed up two pedometer interventions with positive 12-month effects to examine objective PA levels at 3–4 years.

**Methods and findings:**

Long-term follow-up of two completed trials: Pedometer And Consultation Evaluation-UP (PACE-UP) 3-arm (postal, nurse support, control) at 3 years and Pedometer Accelerometer Consultation Evaluation-Lift (PACE-Lift) 2-arm (nurse support, control) at 4 years post-baseline. Randomly selected patients from 10 United Kingdom primary care practices were recruited (PACE-UP: 45–75 years, PACE-Lift: 60–75 years). Intervention arms received 12-week walking programmes (pedometer, handbooks, PA diaries) postally (PACE-UP) or with nurse support (PACE-UP, PACE-Lift). Main outcomes were changes in 7-day accelerometer average daily step counts and weekly time in moderate-to-vigorous PA (MVPA) in ≥10-minute bouts in intervention versus control groups, between baseline and 3 years (PACE-UP) and 4 years (PACE-Lift). PACE-UP 3-year follow-up was 67% (681/1,023) (mean age: 59, 64% female), and PACE-Lift 4-year follow-up was 76% (225/298) (mean age: 67, 53% female). PACE-UP 3-year intervention versus control comparisons were as follows: additional steps/day postal +627 (95% CI: 198–1,056), *p* = 0.004, nurse +670 (95% CI: 237–1,102), *p* = 0.002; total weekly MVPA in bouts (minutes/week) postal +28 (95% CI: 7–49), *p* = 0.009, nurse +24 (95% CI: 3–45), *p* = 0.03. PACE-Lift 4-year intervention versus control comparisons were: +407 (95% CI: −177–992), *p* = 0.17 steps/day, and +32 (95% CI: 5–60), *p* = 0.02 minutes/week MVPA in bouts. Neither trial showed sedentary or wear-time differences. Main study limitation was incomplete follow-up; however, results were robust to missing data sensitivity analyses.

**Conclusions:**

Intervention participants followed up from both trials demonstrated higher levels of objectively measured PA at 3–4 years than controls, similar to previously reported 12-month trial effects. Pedometer interventions, delivered by post or with nurse support, can help address the public health physical inactivity challenge.

**Trial registrations:**

PACE-UP isrctn.com ISRCTN98538934; PACE-Lift isrctn.com ISRCTN42122561.

## Abstract 2

In this follow-up of participants from two completed trials of pedometer-based walking interventions, Tess Harris and colleagues present evidence that increases in physical activity levels seen at 12 months are still present 3-4 years later.

## Author summary

**Why was this study done?:**

- Brisk walking for 30 minutes or more daily on most days of the week can help adults and older adults to achieve moderate-to-vigorous physical activity (MVPA) guidelines for health benefits, yet many do not achieve these levels.
- Previous pedometer-based walking studies have shown positive effects on increased step counts and time in MVPA for up to 12 months.
- For sustained health benefits, increased physical activity levels need to be maintained, yet there is a lack of data from interventions assessed using objectively measured physical activity levels beyond 12 months.

**What did the researchers do and find?:**

- We followed up participants from two primary care 12-week pedometer-based walking trials, including both nurse-supported and postal pedometer arms, to establish whether objectively measured physical activity increases seen at 12 months were sustained at 3–4 years.
- PACE-UP followed up 45–75-year-olds 3 years post-baseline and showed that both nurse-supported and postal pedometer interventions continued to have higher physical activity levels compared to the control group (approximately an extra 28 and 24 minutes/week, respectively, of MVPA in bouts and an extra 670 and 630 steps/day, respectively).
- PACE-Lift results were very similar. In 60–75-year-olds followed up at 4 years post-baseline, the nurse-supported pedometer intervention group spent about 33 minutes/week more time in MVPA in bouts compared to the control group.

**What do these findings mean?:**

- These findings suggest that adult and older adult participants receiving 12-week pedometer-based walking interventions, provided either by post or with nurse support, are still doing more physical activity 3–4 years later.
- Pedometer interventions can help address the public health physical inactivity challenge.

## Introduction

Strong evidence exists for the health benefits of physical activity (PA) for a wide range of conditions [1,2]. Physical inactivity leads to high health service costs [1,3] and is the fourth leading risk factor for global mortality [2]. Adult and older adult guidelines advise ≥150 minutes of moderate-to-vigorous PA (MVPA) weekly, or 75 minutes of vigorous PA, or a combination, in ≥10-minute bouts [1,4], but any increase in PA for inactive people is valuable [5]. Many PA interventions, including pedometer-based interventions, increase PA levels in the short term [6–8]. However, long-term health effects require sustained PA changes [1], and evidence for maintenance is lacking. A meta-analysis of PA interventions (including pedometers) in 55–70-year-olds [8] only identified 2 trials with objective PA data beyond 12 months [9,10]. One showed a significant step-count effect 18 months post-baseline, but only 6 months post-intervention [10]; the other showed a significant increase in step count in the lifestyle group 23 months post-baseline, but only 12 months post-intervention [9]. The meta-analysis authors [8] repeated requests made by previous systematic reviews [11,12] and guidelines [13] for trials to be conducted with longer follow-up periods and objective PA measures.

We previously conducted two pedometer-based walking interventions with adults and older adults, which increased step count and MVPA in bouts at 12 months and provided longer-term follow-up opportunities [14,15]. Both trials recruited postally from primary care and delivered 12-week pedometer-based walking interventions incorporating behaviour change techniques (BCTs) through dedicated practice nurse PA consultations (3 in PACE-UP, 4 in PACE-Lift) or by post (PACE-UP only). PACE-Lift nurse consultations additionally provided feedback on accelerometry findings to participants. PACE-UP recruited 1,023 predominantly inactive 45–75-year-olds. Average baseline daily step count was 7,479 (standard deviation [SD]: 2,671) and average time in MVPA in bouts was 94 (SD: 102) minutes/week. PACE-Lift recruited 298 patients aged 60–75 years. Average baseline daily step count was 7,347 (SD: 2,839) and average time in MVPA in bouts was 92 (SD: 108) minutes/week. Despite age-group and intervention differences, both trials and all intervention groups showed increases in step counts of approximately one-tenth and time in MVPA of over one-third between baseline and 12 months [14,15].

The study aim was to follow up both trial cohorts to examine objectively measured PA levels at 3 years in PACE-UP and 4 years in PACE-Lift. Given the different but overlapping age ranges, interventions that were similar but differed in intensity, and different lengths of follow-up, we analysed the two trials separately, using identical methods, and present the results in parallel.

## Methods

### Study design and participants

#### PACE-UP 3-year follow-up

For the PACE-UP trial 3-year follow-up, London, Hampstead, Research Ethics Committee (UK) granted approval (12L/LO/0219). Written informed consent was gained from all research participants. Trial methods are published [16]; the postal and nurse interventions are summarised in Table 1 and baseline findings are summarised in S1 Table. The handbook and diary are available at www.paceup.sgul.ac.uk/materials. After a 12-month follow-up, 212/322 (66%) of controls were posted a pedometer, handbook, and PA diary and 64/322 (20%) opted for a single nurse appointment, during which they also received these materials. No further follow-up was offered at that point (compared with the trial postal-intervention group, who were telephoned to check that materials had arrived and encouraged to return completed PA diaries). Three-year follow-up collected accelerometry and patient-reported outcome measures (PROMs) by post. To minimise seasonal effects on PA levels, baseline, 12-month, and 3-year outcomes were assessed in the same month. Follow-up ran from October 2015 to November 2016. The protocol, including 3-year follow-up details, is included (S1 Protocol).

**Table 1:** Components of interventions for PACE-UP and PACE-Lift trials.

Component	PACE-UP		PACE-Lift
	Postal	Nurse	Nurse
**Pedometer** Yamax Digi-Walker (Tokyo, Japan) SW-200. Provides step count, requires daily manual recording and resetting	Posted with instructions for use^a^	Given with instructions by nurse at first appointment	Given with instructions by nurse at first appointment
**Dedicated practice nurse PA consultations** (including BCTs)^b^	Not applicable	3 consultations, Week 1, “First Steps” (approximately 30 minutes) Week 5, “Continuing the Changes” (approximately 20 minutes) Week 9, “Building Lasting Habits” (approximately 20 minutes)	4 consultations, Week 1, “First Steps” (approximately 45 minutes) Week 3, “Continuing the Changes” (approximately 30 minutes) Week 7, “Keeping up the Changes” (approximately 30 minutes) Week 11, “Building Lasting Habits” (approximately 30 minutes)
**Accelerometer feedback as part of intervention**	Not applicable	Not applicable	Actigraph GT3X+ (accelerometer) worn for 1 week prior to each nurse appointment. Nurse downloaded accelerometer data during consultation and provided immediate feedback on time spent in sedentary, light, moderate, and vigorous PA levels in relation to activities recorded in PA diary.
**Handbook**^**c**^ (including BCTs)^b^	Posted^a^	Given by nurse at first appointment	Given by nurse at first appointment
**Target setting: step-count goals, PA goals, and use of walking planner**	Blinded pedometer (Yamax DigiWalker CW200) worn for 7 days at baseline to calculate average daily baseline steps, used to set step-count targets. Use of 12-week walking planner. Advised to add 1,500 steps/day, then 3,000 steps/day, to average baseline steps in a graded manner over 12 weeks. “3,000-steps-in-30-minutes” message for PA intensity.	Blinded pedometer (Yamax DigiWalker CW200) worn for 7 days at baseline to calculate average daily baseline steps, used to set step-count targets. Use of 12-week walking planner. Advised to add 1,500 steps/day, then 3,000 steps/day, to average baseline steps in a graded manner over 12 weeks. Targets could be adapted in discussion with nurse. “3,000-steps-in-30-minutes” message for PA intensity.	Nurses discussed appropriate step-count and PA goals with participants based on baseline step count and weekly time in MVPA from accelerometry and any health issues. Participants encouraged to set both step-count and time in MVPA goals, encouraged to “start low and go slow”. Walking planner to help them plan when, where, and with whom they planned to walk. Goals reviewed and reset at each consultation.
**12-week PA and step-count diary** (including BCTs)^b^	Posted^a^ and encouraged to return completed diary to researchers after 12-week intervention.	Given by nurse at first appointment, reviewed by nurse at other appointments, and encouraged to return completed diary to researchers after 12-week intervention.	Given by nurse at first appointment and reviewed at each nurse appointment.

^a^ Researcher telephoned 1 week later to check that supplies had arrived.

^b^BCTs for promoting lasting change in PA levels were provided in nurse consultations, handbooks, and PA diaries; were categorised according to Michies taxonomy [17]; and included goal setting, self-monitoring, feedback, boosting motivation, encouraging social support, addressing barriers, relapse anticipation, etc.

^c^Both PACE-Lift and PACE-UP patient handbooks were adapted from the NHS Health Trainer Handbook [18] and focused on changing PA levels.

Abbreviations: BCT, behaviour change technique; MVPA, moderate-to-vigorous physical activity; NHS, National Health Service; PA, physical activity; PACE-Lift, Pedometer Accelerometer Consultation Evaluation-Lift; PACE-UP, Pedometer And Consultation Evaluation-UP.

#### PACE-Lift 4-year follow-up

For the PACE-Lift trial 4-year follow-up, Oxfordshire Research Ethics Committee C (UK) granted approval (11/H0606/2). Written informed consent was gained from all research participants. Trial methods are published [19]; the intervention is summarised in Table 1 and baseline findings are summarised in S1 Table. After 12-month follow-up, all control group participants were sent a pedometer and instructions; no support was offered. Four-year follow-up collected the accelerometry and PROMs by post, as in PACE-UP. Additionally, for PACE-Lift, the opportunity to meet study participants face to face after postal return of accelerometers and questionnaires was offered to measure anthropometric variables. For consistency with PACE-UP (in which face-to-face contact was not offered), only postal outcomes (accelerometry and PROMs) are reported in this paper. Baseline, 12-month, and 4-year outcomes were assessed in the same month. Follow-up ran from October 2015 to October 2016. The protocol, including 4-year follow-up, is included (S2 Protocol).

### Procedures

Participants who had not withdrawn from either trial by 12 months were eligible. Practices excluded participants who had died, moved away, or developed a terminal illness or dementia. Eligible participants were sent a trial follow-up letter, participant information sheet, consent form, and freepost return envelope. Researchers telephoned participants to discuss any queries. Those interested returned signed consent forms. Participants and researchers were unmasked to intervention allocation.

Instruments, questionnaire measures, and protocols were the same as during the trial. Participants were not asked to increase their PA levels, just to continue usual activity, and thus health limitations did not preclude participation. Participants were instructed to wear the accelerometer (Actigraph GT3X+) on a belt over one hip for 7 consecutive days, from getting up until going to bed. A diary (to record activities) questionnaire and freepost envelope were provided. If accelerometry recording did not result in ≥5 days with ≥540 minutes/day, participants were asked to re-wear monitors (re-wears were required for 20 PACE-UP and 1 PACE-Lift participants). Participants were posted a £10 gift voucher.

The Actigraph GT3X+ measures vertical accelerations in magnitudes from 0.05 to 2.0 g, sampled at 30 Hz, and then summed over a 5-second epoch time period. It can record PA continuously for up to 21 days. Actigraph data were reduced using Actilife software (V6.6.0), ignoring runs of ≥60 minutes of 0 counts [14,15]. Summary variables were as used in the trials [14,15]: step counts, accelerometer wear time, time spent in total MVPA (≥1,952 counts per minute, equivalent to ≥3 metabolic equivalents), time spent in ≥10-minute bouts of MVPA, and time spent sedentary (≤100 counts per minute, equivalent to ≤1.5 metabolic equivalents). Only days with ≥540 minutes of registered time were used. To lessen attrition bias, main analyses of effect included all subjects with ≥1 satisfactory day of recording at 3 (or 4) years.

### Outcomes

Outcomes focussed on changes between baseline measures and follow-up measures at 3 years (PACE-UP) or 4 years (PACE-Lift). For accelerometry, we analysed: (i) change in average daily step count, (ii) change in time spent weekly in MVPA in ≥10-minute bouts, and (iii) change in weekly sedentary time.

Questionnaire PROMs were as for 3- and 12-month outcomes [16,19]: quality of life [20], exercise self-efficacy [21], pain [22], depression [23, 24], and anxiety [23, 25].

### Statistical analysis

Analysis and reporting followed CONSORT guidelines (S1 Protocol, S2 Protocol). Primary analyses were conducted using STATA version 14.0 (StataCorp), with a two-step process to estimate change. In step 1, average daily step counts at 3 years (PACE-UP) or 4 years (PACE-Lift) were computed from a random-effects model, allowing for day of the week and day of wearing the accelerometer as fixed effects and participant as a random effect. In step 2, average daily step count at 3 years (PACE-UP) or 4 years (PACE-Lift) was regressed on estimated baseline average daily step count, with treatment group, age, gender, practice, and month of baseline accelerometry as fixed effects and household as a random effect in a multilevel model. Identical analyses were carried out for MVPA in ≥10-minute bouts, sedentary time, and wear time. Changes in PROMs were estimated using step 2 only.

Primary analyses used 681 (PACE-UP) or 225 (PACE-Lift) participants who provided accelerometry data at 3 or 4 years, respectively. Sensitivity analyses assessed the effect of missingness: (1) multiple imputation methods were used to impute outcome data for those missing at 3 or 4 years, assuming outcomes were missing at random (MAR), conditional on model variables, and using the STATA procedure mi impute, and (2) missing not at random (MNAR) analyses. The purpose of the MNAR analyses was to assess how extreme the missing data needed to be in order to explain away our positive effect estimates. To do this, we used the Stata module rctmiss (Statistical Software Components [SSC] https://ideas.repec.org/s/boc/bocode.html) [26]. Essentially, the rctmiss programme takes as its starting point MAR estimates for all subjects with missing data. It then adds or subtracts steps to the estimates before re-estimating the treatment effects. Thus, we left the control group missing values at their MAR estimates and first subtracted 500 steps/day from the MAR estimates in the treatment groups; we then took a more extreme scenario, in which we subtracted 1,000 steps/day for those in the treatment groups, again leaving the control group missing values at their MAR values.

### Results

**Fig 1:**
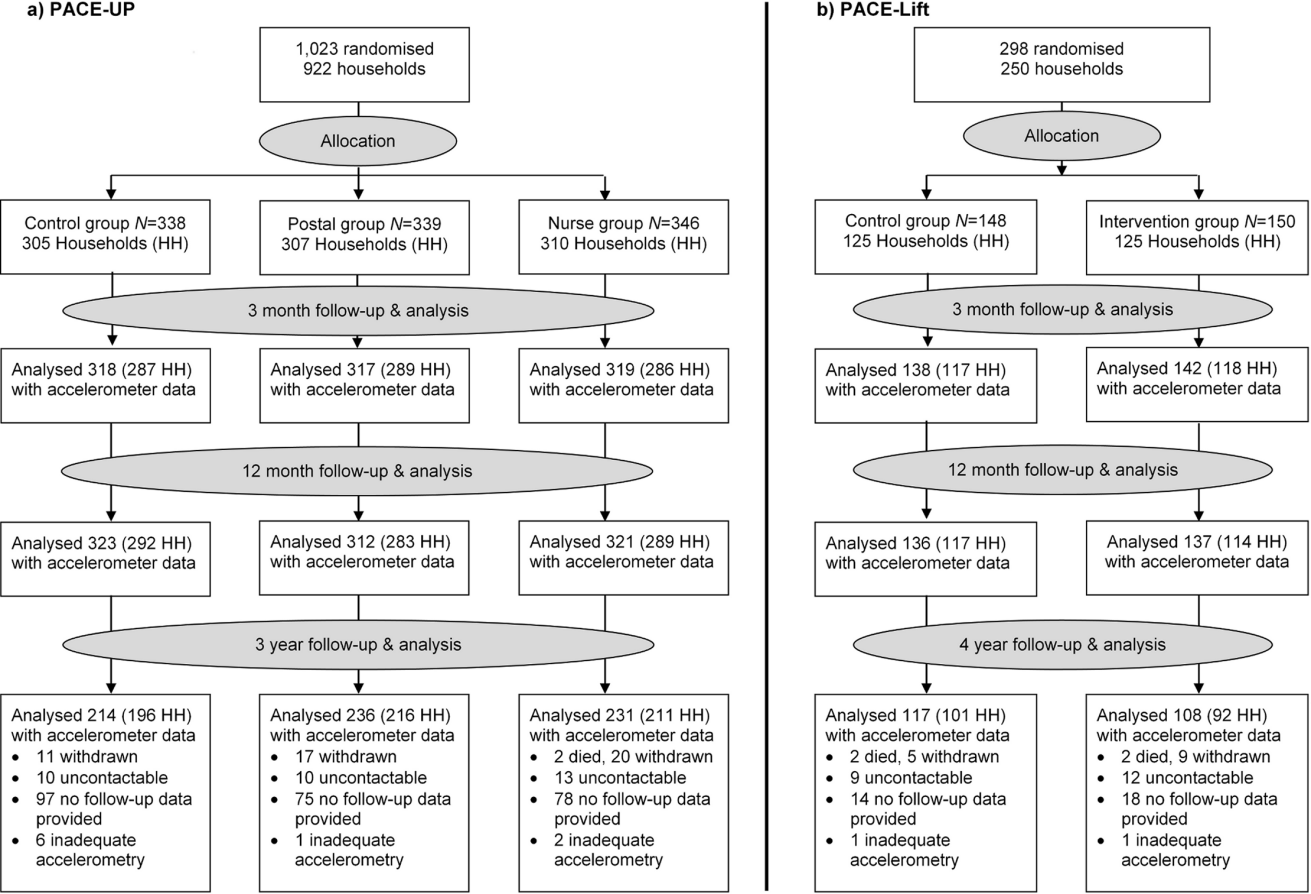
CONSORT diagrams for PACE-UP and PACE-Lift studies. HH, household; PACE-Lift, Pedometer Accelerometer Consultation Evaluation Lift; PACE-UP, Pedometer And Consultation Evaluation-UP.

Of 1,023 PACE-UP participants, 32 withdrew by 12 months, 2 died before the 3-year follow-up, 1 was excluded, and 681 provided ≥1 day of adequate accelerometry data. The 3-year follow-up rate was 69% (681/988), or 67% (681/1,023) of initial trial participants, the mean age was 59 (SD = 7.9), and 64% (438/681) were female. Of 298 PACE-Lift participants, 15 withdrew by 12 months, 2 died before the 4-year follow-up, and 225 provided ≥1 day of adequate accelerometry data. The 4-year follow-up rate was 80% (225/281), or 76% (225/298) of original trial participants, the mean age was 67 (SD = 4.2), and 53% (120/225) were female. The CONSORT diagram (Fig 1) shows 3- and 4-year follow-up data by randomised groups. Ninety-two percent (625/681) in PACE-UP and 93% (209/225) in PACE-Lift provided ≥5 days of accelerometry data at 3 and 4 years, respectively (S2 Table and S3 Table).

**Fig 2:**
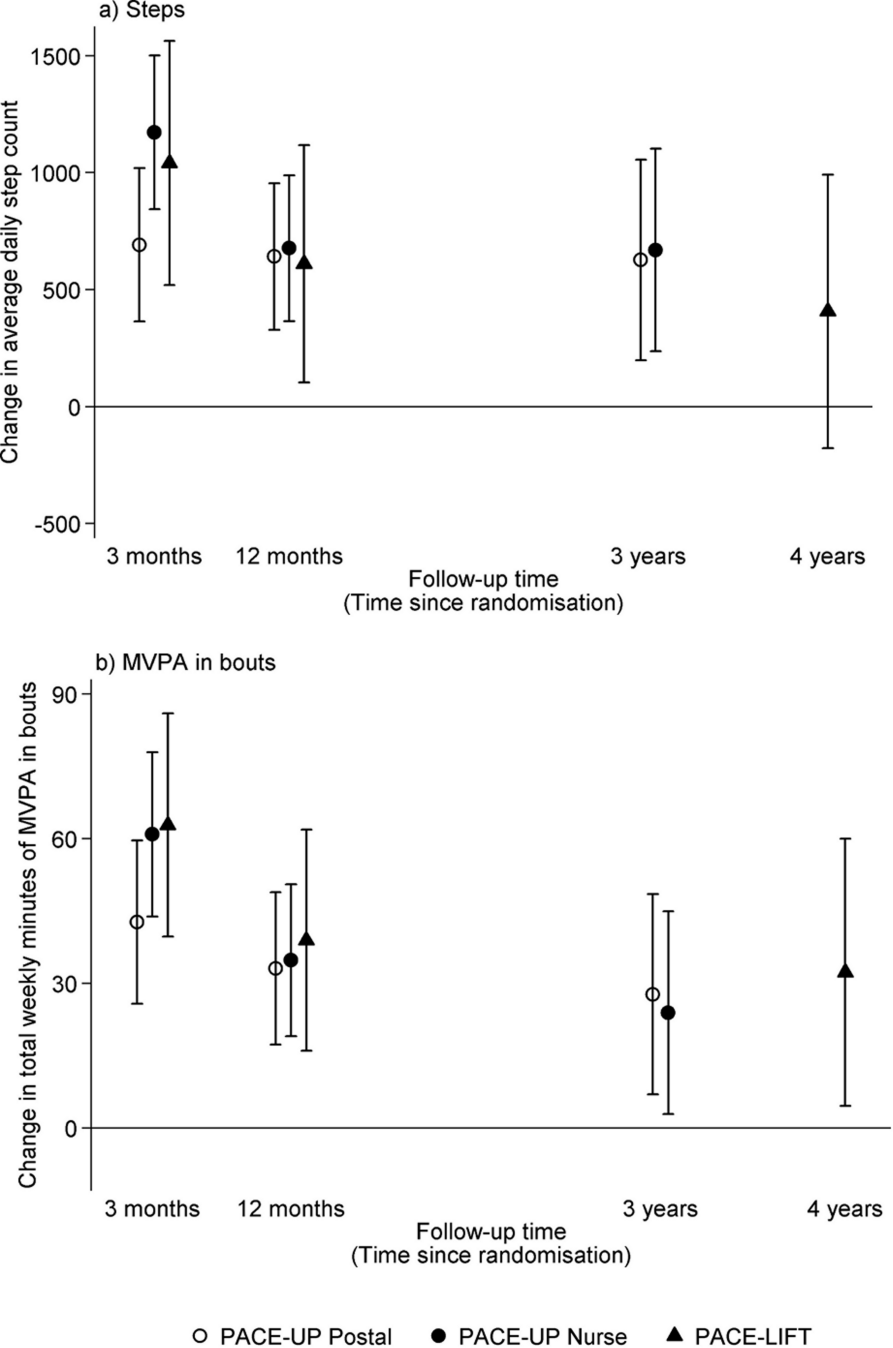
PACE-UP and PACE-Lift studies. **Effect estimates and 95% confidence intervals for change in (a) average daily steps and (b) total weekly minutes of MVPA in bouts at 3 months, 12 months, and 3 years (PACE-UP) and 4 years (PACE-Lift).** Effect sizes, 95% confidence intervals, and *p-*values were obtained from multilevel linear regression models (see Methods). 3 months: *p* < 0.001 for all PACE-UP and PACE-Lift steps and MVPA intervention effects. 12 months: *p* < 0.001 for PACE-UP steps and PACE-UP MVPA; *p* = 0.02 for PACE-Lift steps and *p* < 0.001 for PACE-Lift MVPA. 3 years: *p* < 0.01 for PACE-UP steps and PACE-UP MVPA postal group; *p* = 0.03 for PACE-UP MVPA nurse group. 4 years: *p* = 0.17 for PACE-Lift steps and *p* = 0.02 for PACE-Lift MVPA. MVPA, moderate-to-vigorous physical activity; PACE-Lift, Pedometer Accelerometer Consultation Evaluation-Lift; PACE-UP, Pedometer And Consultation Evaluation-UP.

Accelerometry summary measures are shown for the three PACE-UP groups (S2 Table) and two PACE-Lift groups (S3 Table) at each time point. Fig 2 displays effect estimates for different groups from both trials at all time periods for step counts and time in MVPA in bouts, respectively. Table 2 shows these estimates plus sedentary time and wear time in tabular form. At 3 years in PACE-UP, both intervention groups are doing more steps/day than controls, with no significant intervention group differences: postal +627 (95% CI: 198–1,056); nurse +670 (95% CI: 237–1,102). For PACE-Lift, at 4 years, the intervention group is doing more steps/day than the control group, although the difference is not statistically significant: +407 (95% CI: −177–992). For total weekly MVPA in ≥10-minute bouts (minutes/week), PACE-UP 3-year findings compared with control are as follows: postal +28 (95% CI: 7–49); nurse +24 (95% CI: 3–45). For PACE-Lift at 4 years, the intervention group is still doing significantly more MVPA in bouts (minutes/week) than the control group: +32 (95% CI: 5–60). Effect estimates for both steps per day and MVPA were stable when we limited analyses to subjects with at least 4 days of measurement at follow-up (S4 Table).

**Table 2:** PACE-UP and PACE-Lift studies: Accelerometry outcomes at 3 months, 12 months, and 3 years (PACE-UP) and 4 years (PACE-Lift).

Outcomes	PACE-UP study			PACE-UP study			PACE-Lift study		
	Postal versus Control			Nurse versus Control			Intervention versus Control		
	Effect	95% CI	*p*-value	Effect	95% CI	*p*-value	Effect	95% CI	*p*-value
**Step counts**									
**3 months**	692	(363–1,020)	<0.001	1,173	(844–1,501)	<0.001	1,041	(519–1,563)	<0.001
**12 months**	642	(329–955)	<0.001	677	(365–989)	<0.001	610	(104–1,117)	0.02
**3 years**	627	(198–1,056)	0.004	670	(237–1,102)	0.002			
**4 years**							407	(−177–992)	0.17
**MVPA in ≥10-minute bouts**									
**3 months**	43	(26–60)	<0.001	61	(44–78)	<0.001	63	(40–86)	<0.001
**12 months**	33	(17–49)	<0.001	35	(19–51)	<0.001	39	(16–62)	<0.001
**3 years**	28	(7–49)	0.009	24	(3–45)	0.03			
**4 years**							32	(5–60)	0.02
**Daily sedentary time (minutes)**									
**3 months**	−2	(−12–7)	0.59	−7	(−16–3)	0.16	−1	(−13–11)	0.84
**12 months**	1	(-8–10)	0.82	0	(−9–9)	0.96	0	(−15–15)	0.97
**3 years**	−1	(−12–11)	0.90	−2	(−14–9)	0.69			
**4 years**							7	(−9–23)	0.37
**Daily wear time (minutes)**									
**3 months**	2	(−8–12)	0.69	4	(−6–14)	0.39	14	(0, 28)	0.06
**12 months**	9	(−1–19)	0.08	9	(−1–19)	0.07	5	(−11–22)	0.51
**3 years**	8	(−5–20)	0.23	7	(−6–19)	0.32			
**4 years**							9	(−10–28)	0.35

Analyses using all available data at each follow-up.

PACE-UP study: *N* = 954 at 3 months, 956 at 12 months, and 681 at 3 years. PACE-Lift study: *N* = 280 at 3 months, 273 at 12 months, and 225 at 4 years.

All models include treatment group, practice, gender, age at randomisation, and month of baseline accelerometry as fixed effects and household as a random effect in a multilevel linear regression model. The results shown are the change in each intervention group relative to the change in their control group.

The effect estimates, 95% confidence intervals, and *p*-values were obtained from the model output.

In PACE-UP, the 3-year treatment effects for steps/day were 98% (postal) (627/642) and 99% (nurse) (670/677), respectively, of the 1-year estimates; in PACE-Lift, the 4-year estimate was 67% (407/610) of the 1-year estimate. For minutes of MVPA in 10-minute bouts, PACE-UP estimates were 85% (postal) (28/33) and 69% (nurse) (24/35), respectively, of the 1-year estimates, while the PACE-Lift estimate was 82% (32/39). Neither PACE-UP nor PACE-Lift showed differences between intervention and control groups at 3 and 4 years for sedentary time or daily wear time (Table 2). A PACE-UP subgroup analysis demonstrated similar effects for steps/day in 45–59- and 60–75-year-olds (S1 Fig).

None of the interventions had significant effects on pain, depression, anxiety, or health-related quality of life at 3 or 4 years, consistent with 3- and 12-month findings (S4 Table). In PACE-UP, there was a persistent exercise self-efficacy effect for the nurse group at 3 years (also seen at 3 and 12 months) but not in PACE-Lift at 4 years (S5 Table).

Table 3 presents sensitivity analyses assuming that missing outcome data were MAR, conditional on a variety of predictors; analyses had little impact on the primary outcome step-count effect estimates and do not change interpretation. For the MNAR analyses, we combined both intervention groups in PACE-UP to increase power and simplify presentation; separate analyses give a similar picture. The MNAR analyses (S2 Fig) make a bigger impact for both trials but only when we assume there is a strong differential departure between the non-random effects in control and treatment groups (see solid lines in S2 Fig). For example, when we assume that the missing data in the treatment groups are 1,000 steps below their MAR values while the control group values are at their MAR values, the treatment effects for PACE-UP are no longer statistically significant; but even then, the confidence interval is still largely positive.

**Table 3:** PACE-UP and PACE-Lift studies: Imputation analyses for step counts at 3 years (PACE-UP) and 4 years (PACE-Lift).

Imputation models				PACE-UP study							PACE-Lift study			
		Postal versus Control				Nurse versus Control					Intervention versus Control			
	*N*	Effect	(95% CI)		*p*-value	Effect		(95% CI)		*p*-value	N	Effect	(95% CI)	*p*-value
All participants with follow-up data	681	627	(198–1,056)		0.004	670		(237–1,102)		0.002	225	407	(−177–992)	0.17
Imputed using treatment group, baseline steps, gender, age, practice, month baseline accelerometry	1,023	597	(174–1,020)		0.006	679		(268–1,089)		0.001	298	429	(−152–1,010)	0.15
Imputed using treatment group, baseline steps, gender, age, practice, month baseline accelerometry, baseline deprivation, baseline self-reported pain, and baseline body fat mass^†^	996	634	(211–1,057)		0.003	735		(293–1,178)		0.001	292	437	(−154–1,028)	0.15
Imputed using treatment group, baseline steps, gender, age, practice, month baseline accelerometry, and 12-month steps^††^	965	625	(217–1,033)		0.003	683		(270–1,095)		0.001	280	367	(−181–916)	0.19

Multiple imputations were carried out using the Stata commands mi impute followed by mi estimate in Stata V12, with three different sets of covariates, as listed in the table. The results shown are the change in each intervention group relative to the change in their control group. The effect estimates, 95% confidence intervals, and *p*-values were obtained from the model output.

^†^Imputed values were only available for 996 (PACE-UP) and 292 (PACE-Lift) when including baseline deprivation in the model (PACE-UP NS-SEC and PACE-Lift Index of Multiple Deprivation).

^††^Imputed values only available for 965 (PACE-UP) and 280 (PACE-Lift) when including 12-month steps in the model.

Abbreviations: NS-SEC, National Statistics Socio-economic classification; PACE-Lift, Pedometer Accelerometer Consultation Evaluation-Lift; PACE-UP, Pedometer And Consultation Evaluation-UP.

## Discussion

To our knowledge, these are the first population-based pedometer studies showing effects on objectively measured PA levels more than 12 months post-intervention. Compared to controls, intervention participants followed up from both PACE-UP and PACE-Lift trials showed significant increases in MVPA in bouts at 3 and 4 years of approximately an extra 30 minutes weekly, with no difference between intervention groups in PACE-UP (as was also found at 12 months). PACE-UP showed a significant step-count increase of approximately 650 steps/day; PACE-Lift showed a similar but nonsignificant step-count increase. The increases seen in PA levels were similar to those seen at 12 months. No differences were seen in sedentary or wear time.

This work’s main strength is its documentation of longer-term follow-up results beyond 12 months from trials with objective PA data relevant to guidelines. Both trials were based on population-based primary care samples and achieved good follow-up. Sensitivity analyses demonstrated that effect estimates were robust; only extreme assumptions changed interpretation. We presented findings for two trials with overlapping but different age groups and slightly different intervention intensities and follow-up periods. However, the many similarities (recruited postally from primary care; 12-week pedometer-based interventions, including nurse-support arms; accelerometer-assessed main PA outcome measures beyond 12 months) meant there was considerable value in presenting the results together. Age was not an effect modifier in PACE-UP. Despite their differences, both trials show similar consistent long-term increased time in MVPA in bouts for intervention group participants.

The study also had a number of potential limitations. Long-term follow-up data were provided by 76% of PACE-Lift and 67% of PACE-UP original trial participants. Whilst only a small proportion of participants actively withdrew from each trial, reasons for withdrawal were not systematically collected. Whilst losing between a quarter and a third of subjects at follow-up could reduce the generalizability of the findings, we have directly addressed the risk of attrition bias through sensitivity analyses using appropriate imputation methods, and this gave robust results. Participants and researchers were unmasked to group; however, PA outcomes were assessed objectively by accelerometry, and participants were blind to measurements. Participants might have tried harder with PA when monitored, but this would also have affected controls and would have been reduced by using a 7-day data collection protocol [6]. Also, the intervention groups increased their MVPA in ≥10-minute bouts, implying that participants made changes as advised. Whilst the Actigraph accelerometer provides valid estimates of time spent in different intensity levels, including MVPA [27], any waist-mounted activity monitor may underestimate upper body movement, such as weight training and carrying heavy loads [28]; it also underestimates cycling and did not measure swimming. However, crucially, accelerometers are most sensitive to ambulatory activities such as walking, which was the main intervention component of both trials. A further potential limitation is that minimal interventions were offered to both trial control groups after 12-month follow-up. However, this contamination would tend to weaken intervention effects, so the existence of differences in PA levels at 3 and 4 years is an important positive finding and helps us to understand the additional support required for a successful postal intervention.

This paper provides novel, important evidence on sustained effects of pedometer-based walking interventions on objectively measured PA levels. A recent systematic review of the effectiveness of behavioural interventions in increasing PA at 12–36 months [8] identified two studies that provided objectively measured outcomes beyond 12 months [9,10]. We identified two more recent studies using a similar search strategy [29,30]. In reviewing these studies, several issues emerge. First, interventions differed dramatically in duration, intensity, and resources needed—particularly important when considering cost-effectiveness. Second, studies reported follow-up length post-baseline, not post-intervention; maintenance of effects is defined by the latter. None of the four studies provided outcomes more than 12 months post-intervention: one was 6 months post-intervention [10], two were ongoing at the point of assessment [29, 30], and the final one was 12 months post-intervention [9]. Our two studies thus provide the first clear evidence of efficacy for pedometer-based interventions at 33 months and 45 months post-intervention, providing the type of evidence from PA interventions recently called for [8,11,13]. The simplicity of our postal intervention makes it likely to be more cost-effective than more intensive interventions, and the PACE-UP trial cost-effectiveness analyses at 12-month follow-up demonstrated this [14,31].

Our findings support guidance to promote pedometers alongside support for goal setting, self-monitoring, and feedback [32]. However, it is important to consider which factors in pedometer-based interventions are important for success. Both PACE-UP and PACE-Lift included a pedometer, step-count diary, and patient handbook, including BCTs and practice nurse PA consultations [16,19]. Despite PACE-Lift providing a more intensive nurse intervention than PACE-UP, both trials delivered similar effects on PA outcomes at 12 months [14,15] and at 3 or 4 years. Additionally, nurse and postal interventions in PACE-UP achieved similar outcomes at 12 months [14] and 3 years. These findings confirm that shorter, simpler interventions can be equally effective [8,33]. Systematic reviews suggest that individual tailoring, personalised activity goals, and using a step-count diary are important [6,8]; all interventions from both trials provided these elements. That the minimal postal interventions given to both trial control groups at 12 months were not effective at increasing PA levels suggests that the additional support given to the original PACE-UP trial postal arm (follow-up telephone call after a week and encouragement to return completed PA diary after 3 months) was important for this group’s success. The original postal group also had step-count targets set based on baseline blinded pedometer use and received the intervention when they had just been recruited to the trial, so they may have been more motivated. These factors may also have been important to the trial postal intervention’s success. PA guidelines stress the importance of increasing time in MVPA [1,4] rather than just steps. Both of our interventions addressed this: PACE-Lift by nurse feedback on PA intensity from accelerometers [19] and PACE-UP by the “3,000-steps-in 30-minutes” [34] advice given to nurse-support and postal arms [16]. Both trials were effective at increasing MVPA in bouts for all intervention groups at all outcome assessments: 3 and 12 months [14,15] and now at 3 years (PACE-UP) and 4 years (PACE-Lift).

We took the effect estimates from the simplest intervention (PACE-UP postal) to estimate long-term health benefits. Based on a systematic review that quantified the strength of association between walking and coronary heart disease [35], a 28 minutes per week increase in MVPA in bouts seen in the postal group at 3 years should reduce coronary heart disease risk by approximately 4% (95% CI: 3–5%) (see S1 Text). A cohort study that related pedometer steps to mortality [36] allowed us to estimate that a sustained increase of 627 steps/day in the postal group at 3 years should lead to a decrease in all-cause mortality of approximately 4% (95% CI: 1–5%) (see S1 Text).

Whilst environmental and policy interventions are urgently required to address the global inactivity challenge [37], individual PA behaviour change interventions are also important. The sustained effects seen on objective PA outcomes at 3 years for the lower cost postal intervention suggest that this would be an effective and cost-effective [31] intervention to roll out. Minimal support is also required to check that materials have arrived and to encourage return of completed PA diaries but need not be face to face or delivered by a healthcare professional. We are currently conducting implementation work (PACE-UP Next Steps) exploring reach, retention, and ease of adoption in primary care recruiting via postal and face-to-face routes.

The use of wearables to monitor personal PA levels has dramatically increased, through smartphones, wrist- or body-worn devices, and mobile apps, offering opportunities for increasing PA. The “3,000-steps-in-30-minutes” message captures intensity and could become an important new public health goal [34], with new, easy ways to measure steps. Small short-term studies in adults and older adults demonstrate that mobile PA apps can increase PA self-monitoring [38,39] and engagement in regular PA [38,39] and that body-worn fitness trackers can increase time spent in MVPA [39]. PACE-UP Next Steps is currently testing online resources and a mobile app to support the PACE-UP postal intervention. However, despite new PA monitoring opportunities, it is important not to ignore robust, trial-based evidence on effective and cost-effective pedometer- plus paper-based interventions.

### Conclusion

We previously reported increased PA at 12 months following 12-week pedometer-based walking interventions for adults and older adults recruited through primary care, delivered either by post with minimal support or through nurse-supported PA consultations. The current paper demonstrates that these findings are still present in participants followed up at 3–4 years. The long-term success of these interventions suggests that they could help to address the public health physical inactivity challenge.

## Supporting information

S1 Checklist CONSORT checklist for PACE-UP 3-year follow-up. PACE-UP, Pedometer And Consultation Evaluation-UP.
(DOCX)

S2 Checklist CONSORT checklist for PACE-Lift 4-year follow-up. PACE-Lift, Pedometer Accelerometer Consultation Evaluation-Lift.
(DOCX)

S1 Protocol PACE-UP protocol, including 3-year follow-up. PACE-UP, Pedometer And Consultation Evaluation-UP.
(DOCX)

S2 Protocol PACE-Lift trial protocol, including 4-year follow-up. PACE-Lift, Pedometer Accelerometer Consultation Evaluation-Lift.
(DOCX)

S1 Text Details on cardiovascular and overall mortality risk reduction. (DOCX)

S1 Table PACE-UP and PACE-Lift trial baseline findings. PACE-Lift, Pedometer Accelerometer Consultation Evaluation-Lift; PACE-UP, Pedometer And Consultation Evaluation-UP.
(DOCX)

S2 Table PACE-UP study summary means and standard deviations for accelerometry data at baseline, 3 months, 12 months, and 3 years. PACE-UP, Pedometer And Consultation Evaluation-UP.
(DOCX)

S3 Table PACE-Lift study summary means and standard deviations for accelerometry data at baseline, 3 months, 12 months, and 4 years. PACE-Lift, Pedometer Accelerometer Consultation Evaluation-Lift.
(DOCX)

S4 Table PACE-UP and PACE-Lift studies: effects of limiting analyses to those with at least 4 days of follow-up data at 3 years (PACE-UP) and 4 years (PACE-Lift). PACE-Lift, Pedometer Accelerometer Consultation Evaluation-Lift; PACE-UP, Pedometer And Consultation Evaluation-UP.
(DOCX)

S5 Table PACE-UP and PACE-Lift studies patient-reported outcome measures at 3 months, 12 months, 3 years (PACE-UP), and 4 years (PACE-Lift). PACE-Lift, Pedometer Accelerometer Consultation Evaluation-Lift; PACE-UP, Pedometer And Consultation Evaluation-UP.
(DOCX)

S1 Fig PACE-UP age subgroup analyses for changes in average daily step counts at 3 years. PACE-UP, Pedometer And Consultation Evaluation-UP.
(TIF)

S2 Fig PACE-UP and PACE-Lift trials long-term follow-up missing not at random analyses. PACE-Lift, Pedometer Accelerometer Consultation Evaluation-Lift; PACE-UP, Pedometer And Consultation Evaluation-UP.
(TIF)

## Abbreviations

BCT: behaviour change technique
MAR: missing at random
MNAR: missing not at random
MVPA: moderate-to-vigorous physical activity
PA: physical activity
PACE-Lift: Pedometer Accelerometer Consultation Evaluation-Lift
PACE-UP: Pedometer And Consultation Evaluation-UP
PROM: patient-reported outcome measure
SD: standard deviation
SSC: Statistical Software Components
