# Supplementary material for: Physical activity levels in adults and older adults 3–4 years after pedometer-based walking interventions: Long-term follow-up of participants from two randomised controlled trials in UK primary care
Source: PLoS Med. 2018 Mar 9;15(3):e1002526. doi: 10.1371/journal.pmed.1002526 (PMC5844512; doi:10.1371/journal.pmed.1002526)
Supplement: S2 Checklist — PACE-Lift, Pedometer Accelerometer Consultation Evaluation-Lift. (DOCX) [file pmed.1002526.s002.docx]

**CONSORT 2010 checklist of information for PACE-lift randomised controlled trial 4-year follow-up**

Much of the detail relating to the PACE-Lift trial is reported in the main PACE-Lift trial outcome paper[1] and trial protocol paper[2]. Details are given here for clarity.

| Section/Topic | Item No | Standard Checklist item | Where information is provided in 4-year follow-up paper or other sources |
| --- | --- | --- | --- |
| Title & Abstract | 1a | Identification as a randomised trial in the title YES | Title |
|  | 1b | Structured summary of trial design, methods, results, & conclusions (see CONSORT checklist for abstracts table 2 below). YES | Abstract |
| Background and objectives | 2a | Scientific background and explanation of rationale YES | Introduction paragraphs 1 and 2 |
|  | 2b | Specific objectives or hypotheses YES | Introduction paragraph 3 |
| Trial design | 3a | Description of trial design (such as parallel, factorial) including allocation ratio. YES | Details in main trial outcome paper[1] methods, trial design. |
|  | 3b | Important changes to methods after trial commencement (such as eligibility criteria), with reasons YES | Details in main trial outcome paper[1] methods, changes from planned analysis in protocol. |
| Participants | 4a | Eligibility criteria for participants  YES | Details in main trial outcome paper see [1] methods, participants and trial protocol paper [2]. |
|  | 4b | Settings and locations where the data were collected. YES | Details in main trial outcome paper [1] methods, participants. |
| Interventions | 5 | The interventions for each group with sufficient details to allow replication, including how and when they were actually administered. YES | Details in main trial outcome paper[1] Table 1 and trial protocol paper [2]. Also summarised in Table 1. |
| Outcomes | 6a | Completely defined pre-specified primary and secondary outcome measures, including how and when they were assessed YES | Methods, outcomes. |
|  | 6b | Any changes to trial outcomes after the trial commenced, with reasons | Not applicable |
| Sample size | 7a | How sample size was determined YES | Details in main trial outcome paper[1] Methods, statistical power and analyses and trial protocol paper [2]. Methods, sample size. |
|  | 7b | When applicable, explanation of any interim analyses and stopping guidelines | Not applicable |
| Sequence generation | 8a | Method used to generate the random allocation sequence YES | Details in main trial outcome paper[1] Methods, randomisation and masking. |
|  | 8b | Type of randomisation; details of any restriction (such as blocking and block size) YES | Details in main trial outcome paper[1] Methods, randomisation and masking. |
| Allocation concealment mechanism | 9 | Mechanism used to implement the random allocation sequence (such as sequentially numbered containers), describing any steps taken to conceal the sequence until interventions were assigned. YES | Details in main trial outcome paper[1] Methods, randomisation and masking. |
| Implementation | 10 | Who generated the random allocation sequence, who enrolled participants, and who assigned participants to interventions. YES | Details in main trial outcome paper[1] Methods, randomisation and masking. |
| Blinding | 11a | If done, who was blinded after assignment to interventions (for example, participants, care providers, those assessing outcomes) and how. YES | Details in main trial outcome paper[1]. Methods, randomisation and masking. |
|  | 11b | If relevant, description of the similarity of interventions. YES | Summarised in Table 1. |
| Statistical methods | 12a | Statistical methods used to compare groups for primary and secondary outcomes YES | Details in main trial outcome paper [1]. Methods, statistical analysis |
|  | 12b | Methods for additional analyses, such as subgroup analyses and adjusted analyses. YES | Details in main trial outcome paper [1]. Methods, analysis of outcomes. |
| Participant flow (a diagram is strongly recommended) | 13a | For each group, the numbers of participants who were randomly assigned, received intended treatment, and were analysed for the primary outcome YES | Figure 1b (CONSORT diagram for 4-year follow-up). |
|  | 13b | For each group, losses and exclusions after randomisation, together with reasons YES | Figure 1b (CONSORT diagram for 4-year follow-up). |
| Recruitment | 14a | Dates defining the periods of recruitment and follow-up YES | For main trial see trial outcome paper[1]. Methods, study design and participants.  For 4-year follow-up see Methods, PACE-Lift 4-year follow-up. |
|  | 14b | Why the trial ended or was stopped | Not applicable |
| Baseline data | 15 | A table showing baseline demographic and clinical characteristics for each group YES | Details in main trial outcome paper (Table 2). |
| Numbers analysed | 16 | For each group, number of participants (denominator) included in each analysis and whether the analysis was by original assigned groups YES | CONSORT diagram Figure 1b |
| Outcomes and estimation | 17a | For each primary and secondary outcome, results for each group, and the estimated effect size and its precision (such as 95% confidence interval). YES | Results, Table 2 and Figure 2. |
|  | 17b | For binary outcomes, presentation of both absolute and relative effect sizes is recommended | Not a binary outcome |
| Ancillary analyses | 18 | Results of any other analyses performed, including subgroup analyses and adjusted analyses, distinguishing pre-specified from exploratory. YES | Sensitivity analyses assessed the effect of missing data at 3 years, (Methods, statistical analysis and results Table 3 & S2 Figure). |
| Harms | 19 | All important harms or unintended effects in each group (for specific guidance see CONSORT for harms) YES | For main trial see trial outcome paper[1] Results, Table 5. |
| Limitations | 20 | Trial limitations, addressing sources of potential bias, imprecision, and, if relevant, multiplicity of analyses.  YES | Details in main trial outcome paper[1] Discussion, study strengths and limitations.  Details relating to 4-year follow-up, Discussion (paragraph on strengths and limitations). |
| Generalisability | 21 | Generalisability (external validity, applicability) of the trial findings. YES | Details in main trial outcome paper[1]  Relating to 4-year follow-up, Discussion, implications. |
| Interpretation | 22 | Interpretation consistent with results, balancing benefits and harms, and considering other relevant evidence. YES | Relating to 4-year follow-up, Discussion, comparison with other studies. |
| Registration | 23 | Registration number and name of trial registry YES | At end of abstract. |
| Protocol | 24 | Where the full trial protocol can be accessed, if available  YES | Published trial protocol paper [2]. For 4-year follow-up details, protocol approved by ethics available as S2 Text. |
| Funding | 25 | Sources of funding and other support (such as supply of drugs), role of funders YES | Included in PLOS Medicine online funding declaration submission |

**Table 2: CONSORT checklist for abstracts**

| Item | Standard Checklist item | Included in main trial outcomes paper abstract (S2 Text) [1] |
| --- | --- | --- |
| Title | Identification of study as randomised. | YES |
| Trial design | Description of the trial design (e.g. parallel, cluster, non-inferiority) | YES |
| Methods |  |  |
| Participants | Eligibility criteria for participants and the settings where the data were collected. | YES |
| Interventions | Interventions intended for each group. | YES |
| Objective | Specific objective or hypothesis. | YES |
| Outcome | Clearly defined primary outcome for this report. | YES |
| Randomization | How participants were allocated to interventions. | YES |
| Blinding (masking) | Whether or not participants, care givers, and those assessing the outcomes were blinded to group assignment. | YES |
| Results |  |  |
| Numbers randomized | Number of participants randomized to each group. | YES |
| Numbers analysed | Number of participants analysed in each group. | YES |
| Outcome | For the primary outcome, a result for each group and the estimated effect size and its precision. | YES |
| Harms | Important adverse events or side effects | YES |
| Conclusions | General interpretation of the results. | YES |
| Trial registration | Registration number and name of trial register. | YES |
| Funding | Source of funding. | YES |

**References:**

1. Harris T, Kerry SM, Victor CR, Ekelund U, Woodcock A, Iliffe S, et al. A primary care nurse-delivered walking intervention in older adults: PACE (pedometer accelerometer consultation evaluation)-Lift cluster randomised controlled trial. PLoS Med. 2015;12(2):

2. Harris T, Kerry S, Victor C, Ekelund U, Woodcock A, Iliffe S, et al. Randomised controlled trial of a complex intervention by primary care nurses to increase walking in patients aged 60-74 years: protocol of the PACE-Lift (Pedometer Accelerometer Consultation Evaluation - Lift) trial. BMC Public Health. 2013;13.
