# Supplementary material for: Physical activity levels in adults and older adults 3–4 years after pedometer-based walking interventions: Long-term follow-up of participants from two randomised controlled trials in UK primary care
Source: PLoS Med. 2018 Mar 9;15(3):e1002526. doi: 10.1371/journal.pmed.1002526 (PMC5844512; doi:10.1371/journal.pmed.1002526)
Supplement: S2 Protocol — PACE-Lift, Pedometer Accelerometer Consultation Evaluation-Lift. (DOCX) [file pmed.1002526.s004.docx]

**Protocol for the four year follow-up of the PACE-Lift trial practice nurse delivered pedometer-based walking intervention in 60-75 year olds**

**PACE-Lift +4 (Pedometer Accelerometer Consultation Evaluation - Lift) trial follow-up**

**Protocol version number 2.0 Date 15/05/2015**

**Proposed starting date: 01/07/2015**

**Proposed end date: 31/03/2017 Proposed duration: 21 months**

**Principal Investigator:**

Dr Tess Harris, Reader in Primary Care, Population Health Research Institute, St George’s University of London, SW17 ORE. Tel: 0208 725 2791 email: [tharris@sgul.ac.uk](mailto:tharris@sgul.ac.uk)

General Practitioner, Sonning Common Health Centre, Oxfordshire, RG49SW. Tel: 0118 9722188. Email: [tess.harris@nhs.net](mailto:tess.harris@nhs.net)

**Co-investigators:**

Professor Derek Cook, Professor of Epidemiology, Population Health Research Institute, St George’s University of London, SW17 ORE. Tel: 0208 725 5490. Email: [d.cook@sgul.ac.uk](mailto:d.cook@sgul.ac.uk)

Mrs Sally Kerry, Reader in Medical Statistics, Pragmatic Clinical Trials Unit, Queen Mary’s University of London, E12AT. Tel: 0207 8822515 Email: [s.m.kerry@qmul.ac.uk](mailto:s.m.kerry@qmul.ac.uk)

Professor Christina Victor, Professor of Gerontology and Health Services Research, Brunel University, London UB8 3PH. Tel: 01895 268730. Email [Christina.victor@brunel.ac.uk](mailto:Christina.victor@brunel.ac.uk)

Professor Ulf Ekelund, Professor of Physical Activity Epidemiology, Norwegian School of Sports Science, Oslo, Norway. Email: [ulf.ekelund@nih.no](mailto:ulf.ekelund@nih.no)

Professor Peter Whincup, Professor of Epidemiology, Population Health Research Institute, St George’s University of London, SW17 ORE. Tel: 0208 725 5577. Email: [p.whincup@sgul.ac.uk](mailto:p.whincup@sgul.ac.uk)

Mrs Carole Beighton, Research Associate, Health Care Faculty, St George’s University of London, SW17 ORE. Email [c.beighton@sgul.kingston.ac.uk](mailto:c.beighton@sgul.kingston.ac.uk)

Professor Michael Ussher, Professor of Health Psychology Tel 0208 725 5605 [mussher@sgul.ac.uk](mailto:mussher@sgul.ac.uk)

**Host institution for the award:**

Oxfordshire Primary Care Commissioning Group. Mrs Dot Powers, Research Manager & Governance Officer, Oxfordshire Primary Care Trust, Oxford OX4 2LH. Tel: 01865 336800. email: [dot.powers@oxfordshireccg.nhs.net](mailto:dot.powers@oxfordshireccg.nhs.net)

**Sponsor:**

St George’s University of London, Dr Deborah McCartney, Clinical Research Governance Officer, Cranmer Terrace Tooting, London, SW17 ORE. Tel: 0208 7250892. Email [dmccartn@sgul.ac.uk](mailto:dmccartn@sgul.ac.uk)

**Funder:**

This work is unfunded and is supported by research reserves within the Population Health Research Institute, SGUL.

**Signature Page**

**Chief Investigator:**

Dr Tess Harris **
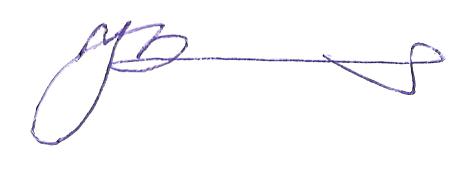
** Date 21/05/2015

**Contents Page**

1. **Summary 4**
2. **Background and rationale 5**
3. **Study objectives and purpose 6**
4. **Study design 7**
5. **Safety 10**
6. **Statistics 10**

*Sample size*

*Outcome measures*

*Statistical analysis*

*Sensitivity analyses for missing data*

1. **Data handling and record keeping 11**

*Confidentiality & data protection*

*Maintenance of study documents*

*Record retention & archiving*

1. **Ethics, compliance & clinical governance 12**

*Ethical considerations*

*Compliance*

*Clinical governance issues*

1. **Monitoring procedures 12**

*Study oversight committees: study management group, study steering committee*

*Audit and inspection*

1. **Finance and insurance 13**

*Contracts & Financial Management*

*Insurance and indemnity arrangements*

1. **Publication and dissemination policy 13**
2. **References 14**

**1.Summary**

**Background**

Physical activity (PA) is a key determinant of health and well-being in older people and reduces mortality. Older adults are advised to accumulate at least 150 minutes of moderate to vigorous intensity PA (MVPA) weekly, which can be achieved through brisk walking, but objective PA assessments suggest less than 5% achieve these levels. Long-term health and wellbeing effects require PA changes to be maintained. However, a recent meta-analysis of PA trials in older people found a limited evidence base beyond 12 months, and concluded that randomised controlled trials with longer follow-up and with objective PA measures are needed. In the PACE-Lift trial a primary care nurse delivered pedometer-based walking intervention increased both time spent in MVPA in at least 10 minute bouts (by 63 minutes/week 95% CI 40-87) and steps (by 1037 steps/day 95% CI 513-1560) in 60-75 year olds, with persistent differences between intervention and control groups at 12 months. This is the first population-based trial to objectively measure time spent in MVPA, with good recruitment for a primary care PA trial (30%) and only 6% loss to follow-up at 12 months. Participant feedback strongly supported future follow-up. The PACE-Lift trial therefore offers an ideal opportunity to study long term follow-up of an individually tailored PA intervention within an NHS setting, in a population-based cohort using objective PA assessment.

**Research questions to be addressed by this research:**

Does a primary care nurse delivered pedometer-based walking intervention have an effect on older peoples’ physical activity levels (step-count, time in MVPA and sedentary time) anthropometric measures, psychosocial wellbeing, physical health, disability or health service use at 4 year follow-up?

**Plan of investigation:**

The design is 4 year follow-up of the existing PACE-Lift trial cohort. Due to important seasonal effects on PA levels, participants will be assessed at the same time of year as their baseline and 12 month assessments, so follow-up will be conducted over 12 months, starting October 2015. The setting is three Berkshire and Oxfordshire general practices. The target population is 298 PACE-Lift trial participants from these practices (aged 60-75 years when recruited Oct 2011-Oct 2012). The follow-up will include: objective assessment of usual PA levels for 7 days using an accelerometer; anthropometry (body mass index, body fat, waist circumference); patient reported outcomes (self-reported PA levels, general health, disability, pain, falls, fractures, EQ5D, depression, anxiety, loneliness); data from GP and hospital (HES) records over trial follow-up period (number of consultations, new diagnoses, referrals, A&E attendance, hospital admissions). The study duration is 21 months to allow for study set-up, practice liaison, checking patient lists before patient follow-up and download of GP data, statistical analysis of follow-up and GP data, and report writing after patient follow-up is completed.

**Potential benefits to patients and the NHS:**

We will assess whether the intervention helped people to make lasting PA changes and if so whether this has led to long-term health benefits by assessment of anthropometry, physical health, disability, psychosocial variables, quality of life and health service use. These findings will help inform national and international guidance on interventions to increase physical activity.

**2. Background and rationale**

Physical activity (PA) is a key determinant of older peoples’ health and reduces the risk of over 20 diseases and conditions, leads to improved function, quality of life and emotional well-being and reduces overall mortality[1]. Physical inactivity is a major cost burden on health services[1]. Older adults are advised to be active daily and to accumulate at least 150 minutes of moderate to vigorous intensity PA (MVPA) weekly, in bouts of 10 minutes or more[1]. Regular walking is the commonest form of PA for older adults and walking at a moderate pace (3 miles (5km)/hour) qualifies as moderate intensity[2]. Walking is overestimated by self-report[3] and despite most older adults being capable of walking[4], objective PA assessment using accelerometry suggest that less than 5% achieve recommended PA levels[5,6].

Increasing older people’s PA, including walking, is challenging. Behaviour change techniques (BCTs) (e.g. goalsetting, self-monitoring) are more effective than health education alone[7]. Best practice includes gradually increasing to moderate intensity, incorporating PA into daily routines (e.g. walking) and monitoring intensity[7]. A systematic review, including studies on older people, demonstrated that interventions tailored to people’s needs and delivered at the individual or household level can encourage walking[8]. Primary care provides an ideal context for PA interventions; it allows population-based sampling of healthy older people, practice nurse involvement and offers continuity of care, with many chronic diseases being indications for increasing PA. NHS Health Checks include adults up to age 74 and incorporate brief advice on increasing PA, often by primary care nurses[9]. Pedometers provide direct feedback on step-counts; accelerometers need computer analysis, but also record PA intensity. Two systematic reviews found pedometer users increased steps/day by 2000-2500, but trials included few older adults, were mainly based on volunteers or patients with specific conditions rather than population based, and had mainly short follow-up, of three to six months[10,11]. Two more recent older adult population based primary care trials, showed significant step-count increases at three months[12,13] and six months[13] but did not report on MVPA[12,13]. Systematic reviews of PA interventions in middle aged to older adults generally (not specifically pedometers) have shown moderate effects of interventions on PA behaviour in the short-to-midterm[14][15] with the most effective interventions including professional guidance[14] and behavioural interventions[14].

Crucially, long-term health and wellbeing effects require PA changes to be maintained, yet few trials in older people provide evidence of this. A systematic review and meta-analysis of behavioural interventions to increase physical activity in older adults (aged 55 to 70 years) examined longer term effects (12 to 36 months)[16]. 21 individual trials were included, six used objective PA assessment (five used pedometers deriving step-count and one used an accelerometer deriving vector magnitude), but none objectively measured MVPA and none reported on cost-effectiveness[16]. Interventions to promote PA were effective at 12 months, both for step-count and self-report PA. Few trials had data beyond 12 months and no intervention effect was seen at 24 months (4 trials with self-report data). Interventions which involved individual tailoring, including personalised step-count goals, were most effective and targeting specific activities, such as walking, was more effective than targeting generic PA in this age group. They concluded that the current evidence base is very limited beyond 12 months and therefore more RCTs with longer follow-up and objective PA measures are needed[16]. Two studies not included in the systematic review have reported long-term PA intervention effects in older people: a walking intervention with self-reported PA outcomes at 5 years[17] and a strength training programme assessing long-term muscle performance at 7 years[18]. Neither provide objective long-term PA data, but both suggest that PA interventions in this age group can have lasting effects to offset age related declines.

In the PACE-Lift trial, primary care nurses delivered an individually tailored pedometer-based walking intervention to 60-75 year olds[19]. The intervention increased both time spent in MVPA in at least 10 minute bouts (63 minutes/week 95% CI 40-87) and steps (1037 steps/day 95% CI 513-1560) at 3 months, and differences between intervention and control groups persisted at 12 months[20]. Ours is the first population-based trial to objectively measure time spent in MVPA and we had good recruitment for a primary care PA trial (30%), with only 6% loss to follow-up at 12 months. Participant feedback strongly supported future follow-up. The PACE-Lift trial was designed to provide lasting effects and several features of the intervention suggest that long-term effects are plausible or likely: there were sustained differences between the groups at 12 months (9 months after the intervention ceased); we based the intervention on increasing walking which allows increase in both step-counts and PA intensity and can easily be built into daily lives[7,8,16]; we used accelerometers to show individuals’ their exact PA at baseline, which allowed individual tailoring of advice[8,16] and we used measured change in PA levels, both intensity and step-counts as a visible feedback cue; we included professional guidance and support[14]; and finally we used behaviour change techniques that encouraged not only change in initial behaviour but in ability to overcome barriers, anticipate relapse and build long-term habits[7][14]. The PACE-Lift trial thus offers an ideal opportunity to study long-term four year follow-up of an individually tailored PA intervention in an NHS setting which produced a clear 12 month effect. The planned follow-up study is population-based and uses objective PA assessment. We will be able to assess whether the intervention helped people to make lasting PA changes and whether this has led to long-term health benefits (anthropometry, physical health, disability, pain, psychosocial variables, quality of life and health service use).

1. **Study objectives and purpose**

The principal research question that this work will address is:

Does a primary care nurse delivered pedometer-based walking intervention have an effect on older peoples' physical activity levels at 4 year follow-up (3 years and 9 months after intervention ceased)?

This will be assessed objectively by accelerometer measurement of usual physical activity levels over 7 days at 4 year follow-up (average daily step-count, time in at least moderate-to-vigorous physical activity (MVPA) in bouts of at least 10 minutes, time spent sedentary) and comparing these to baseline measures, to assess change for each individual.

The secondary research questions that this work will address are:

Does a primary care nurse delivered pedometer-based walking intervention have an effect on older peoples' body size measures, psychosocial wellbeing, physical health, function, disability and health service use at 4 year follow-up (3 years and 9 months after intervention ceased)?

These research questions will be answered in the following ways:

1. Body size measures by assessing change in body mass index, waist circumference, body fat since baseline

2. Psycho-social wellbeing by assessing changes in depression, anxiety and quality of life scores and loneliness since baseline.

3. Physical health, function and disability by assessing change in questionnaire measures of general health, function and disability since baseline.

4. Health service use by assessing the following over the 4 year follow-up period (for those giving consent to access to their records): primary care computerised record data on consultations; medically attended falls; fractures; new diagnoses; referrals; A&E attendances; and hospital admissions.

**4.** **Study design**

4 year follow-up of the PACE-Lift RCT cohort with intervention and control arms.

**Setting:** Three general practices in Oxfordshire and Berkshire, UK.

**Population:** 298 participants (150 intervention 148 control) from the PACE-Lift trial, aged 60-75 years at randomisation, followed up 4 years post randomisation.

**Part A** below summarises the original PACE-Lift trial. **Part B** outlines the 4 year follow-up study.

**Part A: PACE-Lift trial background details:**

The PACE-Lift protocol is published[19]. Trial outcomes have been published[20]. Key points are given for clarity.

*PACE-Lift trial design:*

A two-arm parallel cluster-randomized trial, randomised by household, compared a complex intervention to increase walking carried out over 3 months, with a usual care control group. The primary endpoint was at 3 months; 12 month follow-up assessed maintenance.

*PACE-Lift trial sample selection and exclusions:*

60-74 year olds registered at three Oxfordshire and Berkshire general practices, who could walk outside and had no contra-indications to increasing PA were eligible. Patients were excluded if they were in residential care or had a condition unsuitable for the intervention[19]. A random sample of 200 eligible households, with either one or two older adults at the same address, was selected per practice. Individuals in these households were mailed an invitation. Further households were randomly selected until 100 individuals per practice were randomised.

*PACE-Lift trial procedures:*

*Intervention group:*

The 12 week complex intervention walking programme has been described[19] and comprises:

1. *Four individually tailored practice nurse PA consultations* based on behavioural change techniques (BCTs) eg. target setting, self-monitoring, building self-efficacy and lasting habits.

2. *Yamax Dig-Walker SW-200 pedometer* given to patient to keep at first nurse visit with instructions. Asked to wear before subsequent nurse visits and record step-counts in PA diary.

3. *Actigraph GT3X+ accelerometer*, participants asked to wear it to record PA for 7 days before nurse visits. The nurse downloaded data at the visit and provided visual feedback to participants on time spent in sedentary,light, moderate and vigorous PA intensities, compared with PA diary recordings.

4. *PACE-Lift patient handbook* to support the 12-week walking programme, including BCTs, used by nurses in consultations, given to patients to keep.

5. *Walking / PA plan* an individual walking /PA plan devised during nurse PA consultations. No universal goal; nurses encouraged steps and time spent walking at moderate intensity PA, particularly in bouts of 10 minutes, to be added incrementally to each individual’s baseline. Discussion of when participants would walk, where and who with.

6. *PA diary* to record PA & step-counts, used with monitors to set goals, monitor progress and aid feedback by relating diary activities to accelerometer recorded PA intensities.

*Control group:*

Usual care from the practice, with no other trial appointments. After 12 month follow-up they were posted a Yamax Digi-Walker SW-200 and simple instructions for use.

*PACE-Lift trial main outcome measures:*

*3m assessment:* Conducted as per baseline, at the participant’s practice, including wearing accelerometer for 7 days for both groups. The primary outcome was change in average daily step-count between baseline and 3 months. The secondary outcome was change in average time spent in MVPA weekly between baseline and 3 months. Both assessed by accelerometry. Ancillary outcomes were changes in depression, anxiety and pain scores, BMI and body fat and adverse events (falls etc).

*12m assessment:* Based on accelerometry and questionnaires, sent and returned by post.

*PACE-Lift trial main results[20]:*

*Participants:* Of 988 patients invited, 298 (30%) were randomized to intervention (150) or control (148) groups. 280/298 (94%) provided accelerometer data with ≥1 day of ≥540 minutes wear-time and were included in the 3m primary analysis. 129/150 (86%) of intervention participants attended all 4 sessions.

*Effect of the intervention on PA at 3 & 12 months:* Average daily step-count increased at 3 months in the

intervention group and decreased in the control group; the between-group difference in change was 1037 (95% CI 513, 1560) steps/day (p<0.001). Time spent in MVPA in ≥10 minute bouts followed the same pattern; the between-group difference was 63 (95% CI 40, 87) min/week (p<0.001). At 12 months corresponding differences were 609 (95% CI 104, 1115) steps/day and 40 (95% CI 10, 70) min/week MVPA in ≥10 min bouts.

*Effect of the intervention on other health-related outcomes and adverse events:* No between-group difference in change in BMI at 3 months, 0.001kg/m2(95% CI -0.17, 0.18). A small between-group difference in fat mass -0.39kg (95% CI -0.85, 0.07) was not statistically significant. No between-group differences in mean scores of depression, anxiety, pain or adverse events at 3 or 12 months.

A post-hoc evaluation of costs and cost-effectiveness, using existing trial evidence, was funded after the main trial had shown evidence of effectiveness. This evaluation is in the final stages of analysis.

**Part B: PACE-Lift 4 year follow-up plans for data collection and analysis**

**Timing and manner of data collection:**

To reduce the effect of seasonal variation in PA we will collect 4 year follow-up data over a 12 month period between Oct 2015-Oct 2016. This will ensure that participants provide PA data on their current usual PA levels in the same month that they provided baseline and 12 month data. The main part of the 4 year follow-up will be carried out by post (accelerometry and questionnaires). There will also be the opportunity for participants to attend their general practice once to meet with the research assistant to have their antthopometric (body size) measurements taken.

**Plans for re-contacting participants:**

When we provided individual 12 month feedback we informed participants about future follow-up plans to look at the long-term effect of the intervention on PA levels and health. We asked for participants’ views on future trial follow-up at the feedback parties held at practices and there was a very positive response. We have contact details for all the 298 PACE-Lift trial participants. At the start of planned follow-up we will contact the 3 practices where the participants were registered at the time of PACE-Lift trial recruitment to check names and contact details of the approximately 100 participants from their practice to identify changes in contact details and any who have died. Prior to mailing out invitations we will ask practice nurses to check through the names of those to be invited in the next 3 months for any deaths that may have occurred and also to identify any participants who have developed terminal illness or severe dementia, where it would be inappropriate to contact them. No other medical exclusions from follow-up are needed, as monitoring will be of usual PA levels. The researcher will send out an invitation to participate in the 4 year follow-up with a patient information sheet and consent form (see below).

**Informed consent:**

Participants will be sent a patient letter, participant information sheet and a consent form through the post. They will have a chance to read these and then will be telephoned by a research assistant over the next few days, so that they can ask any questions that they might have. If they are happy to proceed, they will sign their informed consent and post back the top copy of the document to the researcher at St George’s University of London. If the research assistant feels that the potential participant does not have capacity to give informed consent, they will not be recruited to the follow-up. The consent form will also include separate consent for permission to download GP data on consultations for the 4 year follow-up period of the trial, to link to hospital and A&E data (HES records) and consent to meet with the researcher at the patient’s practice to have body size measurements taken.

**Outcome assessment:**

These will be timed to be 4 years (+/- 1 month) from their original randomisation date. The following data will be collected:

*1. Objective PA assessment* - Participants will be asked to wear the Actigraph GT3X+ accelerometer around their hip on a belt from waking until going to bed (excluding showering or swimming) to monitor their current usual PA levels for 7 days. They will be posted out an accelerometer on a belt, with instructions, having arranged a day with the research assistant when the recording will begin. They will post back the accelerometer and belt on completion of the recording.

*2. Questionnaire data to collect patient reported outcomes* (PROs) (general health, disability (Townsend

score[21]), quality of life (EQ5D[22]), depression score (Geriatric Depression Scale-15[23]), anxiety (FEAR[24]), exercise self-efficacy[25], loneliness[26] falls, fractures, injuries, self-report PA levels (GPPAQ[27], IPAQ[28], modified Zutphen[29]), pain, health service use (self-report GP contact and A&E and hospital admissions over last 4 years). The questionnaire will be posted to participants at the same time as the accelerometer and will be posted back to SGUL with it when completed.

*3. Anthropometric assessment* (weight, BMI, body fat from Tanita scales, waist circumference). These measurements will be taken for those consenting to meet with the research assistant at their practice to have this done.

4. *Health service use outcomes* Downloads of primary care computerised records will be done for those giving consent within the whole study cohort after all 4 year outcome assessments have been completed. Consultations, new diagnoses since trial including medically attended falls, fractures, cardiovascular events (MI, CABG, angioplasty, stroke, TIA) new diabetes, depression, referrals.

**Accelerometer data reduction**

ActiGraph data will be reduced using Actilife software set to ignore runs of ≥60 minutes of zero counts[5,19]. The summary variables for the analysis will be: step-counts; accelerometer wear-time; counts per minute (CPM); time spent in at least moderate-to-vigorous PA (MVPA) (≥1952 CPM, ≥3 METs) activity levels using standard Freedson cut-point[30]; time spent in ≥10-min bouts of MVPA; and sedentary time (<100 CPM).

1. **Safety**

This is a follow-up study of a trial cohort, no further intervention is planned. We will be monitoring participants’usual PA levels and not asking them to increase PA, this will be explained fully in the Participant Information Sheet and by the researcher. There are therefore no reasons to exclude participants on the basis of ill-health (apart from terminal illness where it would be inappropriate to contact them, and severe dementia, where it would not be possible to get informed consent). We will however collect data on medically attended falls over the entire follow-up period for those giving consent for us to access their primary care records.

1. **Statistics**

*Sample size calculation:*

Assume 80% follow-up with primary outcome data at 4 years (238/298) (McAuley achieved 72% at 5 years[17], but we have 92% at 12 months and follow-up at 4 years with primary care registration). The main outcome is change in time in MVPA in ≥10 min bouts. Assuming an intra-class correlation of 0.21, average cluster size of 1.17, standard deviation of 86 minutes/week, we will have 80% power at p=0.05 to detect a difference of 32 minutes/week of MVPA in ≥10 minute bouts between intervention and control groups.

*Primary and secondary outcome measures:*

The primary and secondary outcome measures respectively are: i) change in average weekly time spent in MVPA in ≥10 minute bouts; and ii) change in average daily step-count between baseline and 4 year follow-up for the control versus the intervention group.

*Other outcomes:*

Effect of the intervention on: total time in MVPA; time spent sedentary; anthropometric outcomes (BMI, body fat,waist circumference);PROs (depression, anxiety, disability, falls, pain, loneliness, exercise self-efficacy,QoL); health service use outcomes (primary care consultations, referrals, hospital admissions, fractures,medically attended falls etc).

*Data analysis: main outcomes*

Our primary analysis will be based on analyzing all subjects with ≥1 day of 540 minutes wear-time at 4 years (as per 12 month outcomes). We will fit a multi-level model in STATA using the xtmixed procedure. Level 1 will be day within individual, level 2 will be individual and level 3 household. Minutes of MVPA in ≥10 minute bouts (or steps) on a given day at 48 months will be regressed on day order of wear, day of week, age, gender, treatment group and estimated average daily MVPA in ≥10 minute bouts (or steps) at baseline. By including baseline MVPA (or steps) as a covariate, this will effectively measure change in MVPA in ≥10 minute bouts (or step-count) over the 4 year follow-up period. Estimated average daily MVPA in ≥10 minute bouts (steps) at baseline has been derived by using a similar 3-level model in which daily MVPA (steps) was regressed on day order of wear and day of week.

Sedentary time is likely to be highly inversely correlated with time in MVPA and step-count and will be analysed using identical approaches.

*Data analysis: patient reported and anthropometric outcomes*

The effects of the intervention on change in depression, anxiety, disability, pain, self-efficacy and QoL scores, falls, body mass index, waist circumference and body fat will be estimated using a two-level model in which outcome will be regressed on the same measure at baseline, age, gender, and treatment. Level 1 will be individual, level 2 household.

*Data analysis: health service use outcomes*

We will examine the effect of the intervention on health service use outcomes (GP consultations, A&E

attendances and hospital admissions, although the study will not be powered for differences in these outcomes.

*Sensitivity analyses to account for missing data*

To assess if subjects lost to follow-up, or who failed to record a single adequate day of accelerometry at 4 years, might have introduced bias, we will use the STATA procedure *mi impute* to impute PA at 4 years based on baseline, 3 month and 12 month PA, gender, age, and month of baseline accelerometry. Further analyses will add IMD, self-reported pain and fat mass to the imputation.

1. **Data handling and record keeping**

*Confidentiality and data protection:*

Participants’ personal data will remain confidential and will be handled, processed and destroyed in accordance with the terms of the Data Protection Act. Dr Tess Harris is the data custodian. Only the research assistant and principal investigator employed by SGUL and authorized research monitors from the sponsor (SGUL) will have access to non-anonymised data. They are all bound by their organisation’s policies on data protection. All have a duty of confidentiality, nothing that could reveal patients’ identity will be disclosed outside the practice or St George’s University of London.

Personal contact details (eg addresses, telephone numbers and emails) and the code linking patient identity with their unique study ID number are particularly sensitive information and will be stored separately from other study files, in a password protected document, in a secure password protected St George's University of London network drive. Paper copies (e.g of the consent form) will be kept in a locked filing cupboard in a locked office St George's University of London. Electronic copies of data will be saved directly onto a secure password protected network drive at SGUL which is saved on the UNIX system with secure back-ups. All university computers will be password protected, and files will be further password protected to restrict access to those who need access.

Individuals will be asked specifically for their consent for access to their GP records to download information on reason for and number of general practice consultations, A&E attendances and hospital admissions during the last 4 years. Those participants who give written consent for this will have this information downloaded at the end of 4 year follow-up. This will be done at the same time for all the patients at that practice and will be downloaded onto an encrypted SGUL memory stick and transferred the same day to the secure network drive at SGUL and then removed from the encrypted memory stick.

*Maintenance of study documents:* A study master file with essential documents containing information specific to the follow-up study will be kept in the Population Health Research Institute at St George’s University of London.

*Record retention & archiving:* A copy of patient consent forms will be kept for 5 years after the study has ended before being destroyed. Other personal data that is identified by patient name or address will also be destroyed by 5 years after the study has ended. (This is SGUL policy). Other records from the trial will be archived for 7 years after the trial before being destroyed.

1. **Ethics, compliance and clinical governance**

The study will be submitted to Oxfordshire Research Ethics Committee C, who gave a favourable review to the original PACE-Lift trial(11/H0606/2) ahead of study start date.

The main ethical consideration is the risk of harm to participants, the study has a very low risk of harm, as we are following participants up and monitoring their usual physical activity levels (Section 7: Safety). Other ethical considerations are the way that recruitment and informed consent are handled, so that potential participants are not put under pressure to take part and only those with capacity to consent are recruited (Section 4: Study design) and the way that confidentiality is maintained (Section 7: Data handling and record keeping).

*Compliance:* The trial will be conducted in compliance with the protocol, Good Clinical Practice and regulatory requirements.

*Clinical governance issues:*  National Health Service (NHS) Research & Development (R&D) approval has been sought from Oxfordshire and Berkshire West Primary Clinical Commissioning Groups to cover all the practice sites. We will apply for NHS honorary research contracts with Oxfordshire and Berkshire West PCTs for the research assistant before patient contact.

1. **Monitoring**

*Study Oversight Committees*

*Study Management Group (SMG):* SMG members are TH (chief investigator) SK (statistician) DC (epidemiologist) and the research assistant (RA). Meetings will be held monthly, minutes will be retained in the study master file. The SMG will monitor all aspects of the conduct and progress of the study, ensure the protocol is adhered to and take action as necessary. There will be weekly RA supervision meetings (TH), to ensure day-to-day trial management is on track. TH is the project manager with overall responsibility for ensuring timely achievement of milestones.

*Study Steering Committee:* The Study Steering Committee (SSC) will provide overall trial supervision and ensure that it is being conducted in accordance with Good Clinical Practice. They will monitor study progress, including recruitment, data completeness and losses to follow-up and ensure that there are no major deviations from the study protocol. Membership is the original PACE-Lift trial steering committee (TSC) who have agreed to provide continuing supervision for this study. This includes an independent chair (Professor Janet Peacock, statistican), two independent members representing the clinical area under study (a general practitioner, Professor Denise Kendrick and a specialist in care of older people, Professor Cameron Swift), the Principal Investigator TH, the trial statistician SK and Mr Paul Cann, Chief Executive of Age Concern Oxfordshire, to represent users’ views.

*Audit and inspection:*

The trial documentation will be made available to auditors and inspectors representing the sponsor, host institution and regulatory authorities.

1. **Finance and insurance**

*Contracts & Financial Management:*

As the study is unfunded, no contracts will therefore be needed with our co-investigators from Queen Mary’s University of London, Brunel University, London and University of Oslo, Norway. We will have commitment to study agreements with the practices. Any costs incurred by the practices (practice nurse time for checking lists ahead of us mailing out to participants and room hire at practices to do anthropometric assessments) will be agreed with the practices before they are incurred, and purchase orders will be raised for them. The practices will invoice SGUL for costs and they will be paid from Population Health Research Institute reserve funds, as has been agreed with the Institute director.

*Insurance and indemnity arrangements:* SGUL is be the sponsor for the trial as the main employer of the Chief Investigator Dr Harris. There are no special compensation arrangements for the trial. If a patient is harmed during the research study there are no special compensation arrangements. If a patient is harmed and this is due to someone’s negligence, they may have grounds for a legal action for compensation against St George’s, University of London, but they may have to pay their legal costs. The normal NHS complaints mechanisms will still be available to them.

1. **Publication and dissemination policy**

Stage 1: Research findings submitted for publication in peer reviewed journals (e.g. BMJ, Br J Gen Pract etc) & presented as posters, parallel & possible plenary sessions at academic conferences (eg Society of Academic Primary Care, Society for Social Medicine etc). Results will be fed back to participants, nurses, practices, Oxfordshire CCG & other interested parties locally e.g. Age Concern Oxfordshire, through newsletters & local meetings.

1. **References**

1. Department of Health. Start Active, Stay Active: A report on physical activity for health from

the four home countries' Chief Medical Officers. 2011.

2. Morris JN, Hardman AE: Walking to health. *Sports Med* 1997, 23: 306-332.

3. Tudor-Locke CE, Myers AM: Challenges and opportunities for measuring physical activity in sedentary adults. *Sports Med* 2001, 31: 91-100.

4. Simonsick EM et al: Just get out the door! Importance of walking outside the home for maintaining mobility: findings from the women's health and aging study. *J Am Geriatr Soc* 2005, 53: 198-203.

5. Harris TJ et al: What factors are associated with physical activity in older people, assessed objectively by accelerometry? *Br J Sports Med* 2009, 43:442-450.

6. Joint Health Surveys Unit. Health Survey for England 2008 Physical activity & fitness. 2009.

The NHS Information Centre for health & social care.

7. Cress ME *et al*.: Best practices for physical activity programs and behavior counseling in older adult populations. *J Aging PhysAct* 2005, 13: 61-74.

8. Ogilvie D *et al*.: Interventions to promote walking: systematic review. *BMJ* 2007, 334: 1204.

9. NHS Health Checks Programme. Putting prevention first: NHS Health Checks: Vascular Risk

Assessment and Management Best Practice Guidelines. 2009.

10. Bravata DM *et al*.: Using pedometers to increase physical activity and improve health: a systematic review. *JAMA* 2007, 298: 2296-2304.

11. Kang M et al: Effect of pedometer-based physical activity interventions: a meta-analysis. *Res Q Exerc Sport* 2009, 80: 648-655.

12. McMurdo ME *et al*.: Do pedometers increase physical activity in sedentary older women? A randomized controlled trial. *J Am Geriatr Soc* 2010, 58: 2099-2106.

13. Mutrie N *et al*.: Increasing older adults' walking through primary care: results of a pilot randomized controlled trial. *Fam Pract* 2012.

14. Foster C, Hillsdon M, Thorogood M. Interventions for promoting physical activity. Cochrane

database of systematic reviews. CD003180. 2005.

15. Conn VS, Hafdahl AR, Mehr DR: Interventions to increase physical activity among healthy

adults: meta-analysis of outcomes. *Am J Public Health* 2011, 101: 751-758.

16. Hobbs N *et al*.: Are behavioral interventions effective in increasing physical activity at 12 to 36 months in adults aged 55 to 70 years? A systematic review and meta-analysis. *BMC Med* 2013, 11: 75.

17. McAuley et al: Long-term follow-up of physical activity behavior in older adults. *Health Psychol* 2007, 26: 375-380.

18. Kennis E et al: Long-term impact of strength training on muscle strength characteristics in older adults. *Arch Phys Med Rehabil*2013, 2054-2060.

19. Harris T *et al*.: Randomised controlled trial of a complex intervention by primary care nurses to increase walking in patients aged 60-74 years: protocol of the PACE-Lift (Pedometer Accelerometer Consultation Evaluation -Lift) trial. *BMC Public Health* 2013.

20. Harris T, Kerry SM, Victor CR, Ekelund U, Woodcock A, Iliffe S et al. A primary care nurse-delivered

walking intervention in older adults: PACE (pedometer accelerometer consultation evaluation)-Lift

cluster randomised controlled trial. *PLoS Med* 2015; 12(2):e1001783.

21. McGee MA et al: The Medical Research Council Cognitive Functioning and Ageing Study (MRC CFAS). The description of activities of daily living in five centres in England and Wales. *Age & Ageing* 1998, 27: 605-613.

22. Brooks R: EuroQol: the current state of play. *Health Policy* 1996, 37: 53-72.

23. D'Ath P et al: Screening, detection and management of depression in elderly primary care attenders. I: The acceptability and performance of the 15 item Geriatric Depression Scale (GDS15) and the development of short versions. *FamPract* 1994, 11: 260-266.

24. Krasucki C et al: The FEAR: a rapid screening instrument for generalized anxiety in elderly primary care attenders. *Int J Geriatr Psychiatry* 1999, 14: 60-68.

25. Jette AM *et al*.: Home-based resistance training: predictors of participation and adherence. *Gerontologist* 1998, 38: 412-421.

26. Tunstall J: *Old and Alone*. London: Routledge and Kegan; 1957.

27. Department of Health. General Practice Physical Activity Questionnaire. 2006.

28. Booth ML: Assessment of physical activity: an international perspective. *Res Q Exerc Sport*2000, 71: s114-s120.

29. Caspersen CJ et al: The prevalence of selected physical activities and their relation with coronary heart disease risk factors in elderly men: the Zutphen Study, 1985. *Am J Epidemiol* 1991, 133: 1078-1092.

30. Freedson PS et al: Calibration of the Computer Science and Applications, Inc. accelerometer. *Med Sci Sports Exerc* 1998, 30: 777-781.
