# Supplementary material for: Physical activity levels in adults and older adults 3–4 years after pedometer-based walking interventions: Long-term follow-up of participants from two randomised controlled trials in UK primary care
Source: PLoS Med. 2018 Mar 9;15(3):e1002526. doi: 10.1371/journal.pmed.1002526 (PMC5844512; doi:10.1371/journal.pmed.1002526)
Supplement: S1 Text — (DOCX) [file pmed.1002526.s005.docx]

**S1 Text**

**What is the potential benefit of our intervention on Coronary Heart Disease and All-cause mortality?**

Several systematic reviews have assessed the benefits of walking based on pooling data from cohort studies. Typically, the relative risks (RR) are 0.8 in those who are physically more active compared to those who are much less active. The difficulty of interpreting such analyses is their focus on comparing two extreme groups; the physically active versus those inactive. Zheng et al^1^ recognised the importance of studying the functional form of the dose response effect of walking on coronary heart disease (CHD) risk. They concluded that the risk of CHD decreases as amount of brisk walking increases^1^. Specifically, they concluded that 150 minutes of brisk walking/week reduces the incidence of CHD by 19% (a RR of 0.81 (95%CI 0.77, 0.86); relative risk estimates were similar in both sexes and in older and younger subjects. From this we can estimate (see below*) that the increase of 28 minutes per week in the PACE-UP postal group in our study at 3 years would be expected to reduce CHD risk by 4% (95%CI 3%, 5%) if sustained. In a prospective study assessing the benefits of walking in a free-living population sample, Dwyer et al^2^ found that higher daily step count measured by pedometer was linearly associated with reductions in all-cause mortality. Using the same method (see ** below) we estimate that the 627 increase in steps/day in our postal group at 3 years would result in a 4% (1% to 5%) decrease in mortality.

* From the paper by Zheng et al^1^ we can take the fact that log (Risk) increases linearly with minutes of MVPA and that increasing minutes of MVPA/wk by 150 reduces risk by 19% (a relative risk of 0.81), to estimate that increasing MVPA by 28 minutes per week would result in a relative risk of 0.81^(28/150)^=0.81^(.187)^ (95%CI 0.77^(0.187)^, 0.86^(0.187)^) = 0.96(95%CI 0.95,0.97) i.e. 4% CHD reduction (95%CI 3%,5%).

** From the paper by Dwyer et al 2015 (PLOS ONE 10(11):e0141274)^2^ the adjusted hazard ratio for all-cause mortality associated with an additional 1000 steps was 0.94 (95% CI .90 to .98). Using the same approach as above an increase of 642 steps/day is estimated to reduce risk by 0.94^(627/1000)^=0.94^(.627)^ (95%CI 0.90^(0.627)^, 0.98^(0.627)^) = 0.96(95%CI 0.94,0.99) i.e. 4% CHD reduction (95%CI 3%,5%).

References

1. Zheng H, Orsini N, Amin J, Wolk A, Nguyen VT, Ehrlich F. Quantifying the dose-response of walking in reducing coronary heart disease risk: meta-analysis. Eur J Epidemiol. 2009;24(4):181-92. doi: 10.1007/s10654-009-9328-9 [doi].

2. Dwyer T, Pezic A, Sun C, Cochrane J, Venn A, Srikanth V, et al. Objectively Measured Daily Steps and Subsequent Long Term All-Cause Mortality: The Tasped Prospective Cohort Study. PLoS One. 2015;10(11):e0141274. doi: 10.1371/journal.pone.0141274. PubMed PMID: 26536618; PubMed Central PMCID: PMCPMC4633039.
