# Supplementary material for: Physical activity levels in adults and older adults 3–4 years after pedometer-based walking interventions: Long-term follow-up of participants from two randomised controlled trials in UK primary care
Source: PLoS Med. 2018 Mar 9;15(3):e1002526. doi: 10.1371/journal.pmed.1002526 (PMC5844512; doi:10.1371/journal.pmed.1002526)
Supplement: S1 Table — PACE-Lift, Pedometer Accelerometer Consultation Evaluation-Lift; PACE-UP, Pedometer And Consultation Evaluation-UP. (DOCX) [file pmed.1002526.s006.docx]

**S1 Table. Baseline characteristics of the PACE-UP and PACE-Lift cohorts**

|  | **PACE-UP study (N=1023)** | | **PACE-Lift study (N=298)** | |
| --- | --- | --- | --- | --- |
|  | **n** | **(%)** | **n** | **(%)** |
| **Age at randomisation** |  |  |  |  |
| 45-59 years | 529 | (52%) | 0 |  |
| 60-75 years | 494 | (48%) | 298 | (100%) |
| **Gender:** male | 367 | (36%) | 138 | (46%) |
| **Marital status**: married | 658 | (66%) | 240 | (81%) |
| **Randomised as a couple** | 207 | (20%) | 99 | (33%) |
| **National Statistics Socio-economic Classification (NS-SEC) (current or previous job)** |  |  |  |  |
| Higher managerial, administrative, professional | 574 | (59%) |  |  |
| Intermediate occupations | 250 | (26%) |  |  |
| Routine & manual occupations | 147 | (15%) |  |  |
| **National quintiles of Index of Multiple Deprivation Rank** |  |  |  |  |
| 1-3 (Most deprived) |  |  | 29 | (10%) |
| 4 |  |  | 51 | (17%) |
| 5 (Least deprived) |  |  | 218 | (73%) |
| **Ethnicity** |  |  |  |  |
| White | 790 | (80%) | 290 | (99%) |
| Asian/Asian British | 68 | (7%) | 2 | (0.7%) |
| Black/African/Caribbean/Black British | 101 | (10%) | 1 | (0.3%) |
| Other | 25 | (3%) | 1 | (0.3%) |
| **Current smoker** | 82 | (8%) | 16 | (5%) |
| **General Health**: Very good or good | 819 | (82%) | 260 | (89%) |
| **Chronic diseases** |  |  |  |  |
| None | 381 | (38%) | 91 | (31%) |
| 1-2 | 542 | (54%) | 178 | (60%) |
| ≥3 | 82 | (8%) | 29 | (10%) |
| **Self-reported pain** | 690 | (69%) | 201 | (68%) |
| **Limiting long-standing illness** | 223 | (22%) | 72 | (25%) |
| **Townsend disability score** |  |  |  |  |
| None (0) | 596 | (59%) | 206 | (70%) |
| Slight of some disability (1-6) | 381 | (38%) | 83 | (28%) |
| Appreciable or severe disability (7-18) | 30 | (3%) | 6 | (2%) |
| **HADS depression score**: borderline or high | 111 | (11%) |  |  |
| **HADS anxiety score**: borderline or high | 200 | (20%) |  |  |
| **Geriatric depression score**: high |  |  | 19 | (7%) |
| **FEAR anxiety score**: high |  |  | 44 | (15%) |
| **Exercise self-efficacy score**: low | 315 | (32%) | 71 | (25%) |

|  | **PACE-UP study (N=1023)** | | **PACE-Lift study (N=298)** | |
| --- | --- | --- | --- | --- |
|  | **n** | **(%)** | **n** | **(%)** |
| **Physical characteristics** |  |  |  |  |
| Overweight/obese: BMI ≥25kg/m^2^ | 681 | (67%) | 200 | (67%) |
|  | **Mean** | **(sd)** | **Mean** | **(sd)** |
| Fat mass (kg) | 26 | (11) | 24 | (9) |
| Waist circumference (cm) | 93 | (14) | 96 | (13) |
| **Accelerometry data** |  |  |  |  |
| **Average adjusted baseline step count per day** |  |  |  |  |
| Mean (sd) | 7479 | (2671) | 7347 | (2839) |
| Median (IQR) | 7324 | (5551 to 9115) | 7071 | (5302 to 9172) |
| **Total weekly minutes of moderate to vigorous physical activity (MVPA) in ≥10 minute bouts** |  |  |  |  |
| Mean (sd) | 94 | (102) | 92 | (108) |
| Median (IQR) | 65 | (20 to 133) | 53 | (3 to 140) |
| **Average adjusted daily sedentary time (minutes)** |  |  |  |  |
| Mean (sd) | 615 | (72) | 598 | (65) |
| Median (IQR) | 613 | (563 to 663) | 593 | (554 to 649) |
| **Average adjusted daily wear time (minutes)** |  |  |  |  |
| Mean (sd) | 791 | (79) | 793 | (76) |
| Median (IQR) | 791 | (735 to 847) | 791 | (738 to 847) |

**Footnotes**

Full references for General Health, Chronic Disease Score, self-reported pain, Townsend Disability Score, HADS depression and anxiety scores, Geriatric Depression Score, FEAR anxiety score, Exercise Self-Efficacy Score are given in the trial protocols^1,2^

**References**

1. Harris T, Kerry SM, Victor CR, Shah SM, Iliffe S, Ussher M, et al. PACE-UP (Pedometer and consultation evaluation - UP) - a pedometer-based walking intervention with and without practice nurse support in primary care patients aged 45-75 years: study protocol for a randomised controlled trial. Trials. 2013;14:418. doi: 1745-6215-14-418 [pii];10.1186/1745-6215-14-418 [doi].

2. Harris T, Kerry S, Victor C, Ekelund U, Woodcock A, Iliffe S, et al. Randomised controlled trial of a complex intervention by primary care nurses to increase walking in patients aged 60-74 years: protocol of the PACE-Lift (Pedometer Accelerometer Consultation Evaluation - Lift) trial. BMC Public Health. 2013;13. doi: 10.1186/1471-2458-13-5. PubMed PMID: WOS:000313448900001.
