# Supplementary material for: Physical activity levels in adults and older adults 3–4 years after pedometer-based walking interventions: Long-term follow-up of participants from two randomised controlled trials in UK primary care
Source: PLoS Med. 2018 Mar 9;15(3):e1002526. doi: 10.1371/journal.pmed.1002526 (PMC5844512; doi:10.1371/journal.pmed.1002526)
Supplement: S2 Table — PACE-UP, Pedometer And Consultation Evaluation-UP. (DOCX) [file pmed.1002526.s007.docx]

**S2 Table. PACE-UP study: Summary means and standard deviations for accelerometry data at baseline, 3 months, 12 months and 3 years**

|  | **Control group (mean (sd))** | | | | **Postal group (mean (sd))** | | | | **Nurse group (mean (sd))** | | | |
| --- | --- | --- | --- | --- | --- | --- | --- | --- | --- | --- | --- | --- |
|  | **Baseline** | **3 months** | **12 months** | **3 years** | **Baseline** | **3 months** | **12 months** | **3 years** | **Baseline** | **3 months** | **12 months** | **3 years** |
| **Number of participants** | **338** | **318** | **323** | **214** | **339** | **317** | **312** | **236** | **346** | **319** | **321** | **231** |
| **Number (%) with ≥5 days wear** | 338 | 286 | 300 | 188 | 339 | 282 | 287 | 222 | 346 | 296 | 302 | 215 |
|  | (100%) | (90%) | (93%) | (88%) | (100%) | (89%) | (92%) | (94%) | (100%) | (93%) | (94%) | (93%) |
|  |  |  |  |  |  |  |  |  |  |  |  |  |
| **Daily step count** | 7379 | 7327 | 7246 | 7281 | 7402 | 8086 | 8010 | 7896 | 7653 | 8707 | 8131 | 8131 |
|  | (2696) | (2688) | (2671) | (2721) | (2476) | (3014) | (2922) | (2853) | (2826) | (3206) | (3228) | (3410) |
|  |  |  |  |  |  |  |  |  |  |  |  |  |
| **Total weekly mins of**  **MVPA in ≥10 minute bouts** | 84 | 87 | 89 | 94 | 92 | 136 | 129 | 132 | 105 | 164 | 138 | 138 |
|  | (97) | (101) | (94) | (102) | (90) | (125) | (124) | (124) | (116) | (154) | (141) | (161) |
|  |  |  |  |  |  |  |  |  |  |  |  |  |
| **Total weekly mins of MVPA** | 305 | 325 | 318 | 317 | 305 | 366 | 360 | 349 | 324 | 402 | 365 | 362 |
|  | (151) | (166) | (153) | (159) | (140) | (168) | (168) | (166) | (161) | (186) | (186) | (199) |
|  |  |  |  |  |  |  |  |  |  |  |  |  |
| **Daily sedentary time (mins)** | 613 | 614 | 616 | 615 | 614 | 614 | 617 | 617 | 619 | 613 | 620 | 620 |
|  | (68) | (70) | (72) | (71) | (71) | (74) | (71) | (75) | (78) | (77) | (79) | (69) |
|  |  |  |  |  |  |  |  |  |  |  |  |  |
| **Daily wear time (mins)** | 789 | 795 | 791 | 789 | 787 | 798 | 800 | 798 | 797 | 805 | 807 | 805 |
|  | (73) | (78) | (76) | (78) | (78) | (84) | (80) | (86) | (84) | (85) | (89) | (81) |
|  |  |  |  |  |  |  |  |  |  |  |  |  |
