# Supplementary material for: Physical activity levels in adults and older adults 3–4 years after pedometer-based walking interventions: Long-term follow-up of participants from two randomised controlled trials in UK primary care
Source: PLoS Med. 2018 Mar 9;15(3):e1002526. doi: 10.1371/journal.pmed.1002526 (PMC5844512; doi:10.1371/journal.pmed.1002526)
Supplement: S3 Table — PACE-Lift, Pedometer Accelerometer Consultation Evaluation-Lift. (DOCX) [file pmed.1002526.s008.docx]

**S3 Table. PACE-Lift study: Summary means and standard deviations for accelerometry data at baseline, 3 months, 12 months and 4 years.**

|  | **Control group (mean (sd))** | | | |  | **Intervention group (mean (sd))** | | | |
| --- | --- | --- | --- | --- | --- | --- | --- | --- | --- |
|  | **Baseline** | **3 months** | **12 months** | **4 years** |  | **Baseline** | **3 months** | **12 months** | **4 years** |
| **Number of participants** | **148** | **138** | **136** | **117** |  | **150** | **142** | **137** | **108** |
| **Number (%) with ≥5 days wear** | 148 | 127 | 125 | 105 |  | 150 | 134 | 130 | 104 |
|  | (100%) | (92%) | (92%) | (90%) |  | (100%) | (94%) | (95%) | (96%) |
|  |  |  |  |  |  |  |  |  |  |
| **Daily step count** | 7380 | 6904 | 6872 | 7023 |  | 7314 | 7903 | 7514 | 7297 |
|  | (2988) | (3061) | (2792) | (3055) |  | (2693) | (3194) | (3165) | (3237) |
|  |  |  |  |  |  |  |  |  |  |
| **Total weekly mins of**  **MVPA in ≥10 minute bouts** | 88 | 72 | 75 | 93 |  | 96 | 134 | 118 | 126 |
|  | (113) | (102) | (108) | (110) |  | (104) | (138) | (130) | (141) |
|  |  |  |  |  |  |  |  |  |  |
| **Total weekly mins of MVPA** | 301 | 278 | 285 | 282 |  | 296 | 333 | 319 | 305 |
|  | (169) | (169) | (174) | (169) |  | (154) | (185) | (188) | (180) |
|  |  |  |  |  |  |  |  |  |  |
| **Daily sedentary time (mins)** | 595 | 590 | 588 | 587 |  | 601 | 594 | 593 | 598 |
|  | (66) | (68) | (71) | (71) |  | (65) | (67) | (75) | (65) |
|  |  |  |  |  |  |  |  |  |  |
| **Daily wear time (mins)** | 789 | 776 | 780 | 771 |  | 797 | 796 | 792 | 783 |
|  | (72) | (78) | (80) | (87) |  | (79) | (83) | (87) | (89) |
|  |  |  |  |  |  |  |  |  |  |
