# Supplementary material for: Physical activity levels in adults and older adults 3–4 years after pedometer-based walking interventions: Long-term follow-up of participants from two randomised controlled trials in UK primary care
Source: PLoS Med. 2018 Mar 9;15(3):e1002526. doi: 10.1371/journal.pmed.1002526 (PMC5844512; doi:10.1371/journal.pmed.1002526)
Supplement: S4 Table — PACE-Lift, Pedometer Accelerometer Consultation Evaluation-Lift; PACE-UP, Pedometer And Consultation Evaluation-UP. (DOCX) [file pmed.1002526.s009.docx]

|  | **PACE-UP STUDY** | | | | | | | **PACE-LIFT STUDY** | | | |
| --- | --- | --- | --- | --- | --- | --- | --- | --- | --- | --- | --- |
|  |  | **Postal vs Control** | | | **Nurse vs Control** | | |  | **Intervention vs Control** | | |
|  | **N** | **Effect** | **95% CI** | ***p*-value** | **Effect** | **95% CI** | ***p*-value** | **N** | **Effect** | **95% CI** | ***p*-value** |
|  |  |  |  |  |  |  |  |  |  |  |  |
| **Average daily step count** |  |  |  |  |  |  |  |  |  |  |  |
| At least one day of 540 minutes wear time at 3 or 4 year follow-up | 681 | 627 | (198, 1056) | 0.004 | 670 | (237, 1102) | 0.002 | 225 | 407 | (-177, 992) | 0.17 |
|  |  |  |  |  |  |  |  |  |  |  |  |
| At least four days of 540 minutes wear time at 3 or 4 year follow-up | 658 | 700 | (270, 1130) | 0.001 | 714 | (282, 1147) | 0.001 | 215 | 346 | (-246, 939) | 0.25 |
|  |  |  |  |  |  |  |  |  |  |  |  |
|  |  |  |  |  |  |  |  |  |  |  |  |
| **Total weekly MVPA in ≥10 minute bouts** |  |  |  |  |  |  |  |  |  |  |  |
| At least one day of 540 minutes wear time at 3 or 4 year follow-up | 681 | 28 | (7, 49) | 0.009 | 24 | (3, 45) | 0.03 | 225 | 32 | (5, 60) | 0.02 |
|  |  |  |  |  |  |  |  |  |  |  |  |
| At least four days of 540 minutes wear time at 3 or 4 year follow-up | 658 | 28 | (7, 49) | 0.01 | 24 | (2, 45) | 0.03 | 215 | 35 | (6, 63) | 0.02 |

**S4 Table. PACE-UP and PACE-Lift studies: Effects of limiting analyses to those with at least four days of follow-up data at 3 years (PACE-UP) and 4 years (PACE‑Lift)**

**Footnotes**

Analyses using all available data at each follow-up.

All models include treatment group, practice, gender, age at randomisation, and month of baseline accelerometry as fixed effects and household as a random effect in a multi-level linear regression model. The results shown are the change in each Intervention group relative to the change in their Control group.

The effect estimates, 95% confidence intervals and *p*-values were obtained from the model output.
