# Supplementary material for: Physical activity levels in adults and older adults 3–4 years after pedometer-based walking interventions: Long-term follow-up of participants from two randomised controlled trials in UK primary care
Source: PLoS Med. 2018 Mar 9;15(3):e1002526. doi: 10.1371/journal.pmed.1002526 (PMC5844512; doi:10.1371/journal.pmed.1002526)
Supplement: S5 Table — PACE-Lift, Pedometer Accelerometer Consultation Evaluation-Lift; PACE-UP, Pedometer And Consultation Evaluation-UP. (DOCX) [file pmed.1002526.s010.docx]

**S5 Table. PACE-UP and PACE-Lift studies: Patient-reported outcomes at 3 months, 12 months, 3 years (PACE-UP) and 4 years (PACE-Lift**)

|  |  | **PACE-UP study** | | | | | | |  | **PACE-Lift study** | | |
| --- | --- | --- | --- | --- | --- | --- | --- | --- | --- | --- | --- | --- |
|  |  | **Postal vs Control** | | |  | **Nurse vs Control** | | |  | **Intervention vs Control** | | |
|  |  | **Effect** | **95% CI** | ***p*-value** |  | **Effect** | **95% CI** | ***p*-value** |  | **Effect** | **95% CI** | ***p*-value** |
| **Self-reported pain** | **3 months** | 0.05 | (-0.06, 0.17) | 0.37 |  | 0.05 | (-0.07, 0.16) | 0.41 |  | 0.01 | (-0.15, 0.18) | 0.86 |
|  | **12 months** | 0.05 | (-0.06, 0.17) | 0.35 |  | 0.02 | (-0.10, 0.13) | 0.76 |  | 0.04 | (-0.13, 0.20) | 0.69 |
|  | **3 years** | -0.04 | (-0.17, 0.10) | 0.58 |  | -0.11 | (-0.24, 0.02) | 0.11 |  |  |  |  |
|  | **4 years** |  |  |  |  |  |  |  |  | -0.04 | (-0.22, 0.15) | 0.70 |
|  |  |  |  |  |  |  |  |  |  |  |  |  |
| **HADS anxiety score** | **3 months** | -0.29 | (-0.66, 0.09) | 0.13 |  | -0.27 | (-0.65, 0.10) | 0.16 |  |  |  |  |
|  | **12 months** | -0.21 | (-0.60, 0.18) | 0.28 |  | -0.21 | (-0.60, 0.17) | 0.28 |  |  |  |  |
|  | **3 years** | -0.26 | (-0.75, 0.23) | 0.30 |  | -0.38 | (-0.87, 0.11) | 0.13 |  |  |  |  |
|  | **4 years** |  |  |  |  |  |  |  |  |  |  |  |
|  |  |  |  |  |  |  |  |  |  |  |  |  |
| **HADS depression score** | **3 months** | -0.24 | (-0.55, 0.06) | 0.12 |  | -0.21 | (-0.52, 0.10) | 0.19 |  |  |  |  |
|  | **12 months** | -0.13 | (-0.47, 0.20) | 0.44 |  | -0.02 | (-0.36, 0.32) | 0.91 |  |  |  |  |
|  | **3 years** | 0.01 | (-0.40, 0.43) | 0.95 |  | -0.06 | (-0.47, 0.36) | 0.79 |  |  |  |  |
|  | **4 years** |  |  |  |  |  |  |  |  |  |  |  |
|  |  |  |  |  |  |  |  |  |  |  |  |  |
| **FEAR anxiety score** | **3 months** |  |  |  |  |  |  |  |  | 0.02 | (-0.13, 0.16) | 0.83 |
|  | **12 months** |  |  |  |  |  |  |  |  | 0.01 | (-0.15, 0.17) | 0.88 |
|  | **3 years** |  |  |  |  |  |  |  |  |  |  |  |
|  | **4 years** |  |  |  |  |  |  |  |  | -0.10 | (-0.25, 0.05) | 0.20 |
|  |  |  |  |  |  |  |  |  |  |  |  |  |
| **Geriatric depression score (GDS)** | **3 months** |  |  |  |  |  |  |  |  | -0.22 | (-0.55, 0.12) | 0.20 |
|  | **12 months** |  |  |  |  |  |  |  |  | -0.22 | (-0.59, 0.14) | 0.23 |
|  | **3 years** |  |  |  |  |  |  |  |  |  |  |  |
|  | **4 years** |  |  |  |  |  |  |  |  | -0.20 | (-0.55, 0.16) | 0.27 |
|  |  |  |  |  |  |  |  |  |  |  |  |  |
| **EQ-5D** | **3 months** | -0.01 | (-0.02, 0.01) | 0.60 |  | -0.01 | (-0.03, 0.01) | 0.26 |  | 0.01 | (-0.01, 0.04) | 0.31 |
|  | **12 months** | -0.01 | (-0.03, 0.01) | 0.29 |  | -0.01 | (-0.03, 0.01) | 0.22 |  | -0.01 | (-0.04, 0.02) | 0.55 |
|  | **3 years** | 0.00 | (-0.03, 0.02) | 0.86 |  | 0.00 | (-0.02, 0.03) | 0.80 |  |  |  |  |
|  | **4 years** |  |  |  |  |  |  |  |  | 0.04 | (0.00, 0.08) | 0.08 |
|  |  |  |  |  |  |  |  |  |  |  |  |  |
| **Exercise self-efficacy** | **3 months** | 1.12 | (0.23, 2.00) | 0.01 |  | 2.28 | (1.39, 3.17) | <0.001 |  | 2.52 | (1.14, 3.89) | <0.001 |
|  | **12 months** | 0.62 | (-0.33, 1.57) | 0.20 |  | 1.21 | (0.27, 2.16) | 0.01 |  | 0.06 | (-1.52, 1.63) | 0.94 |
|  | **3 years** | 0.53 | (-0.75, 1.80) | 0.42 |  | 1.75 | (0.46, 3.03) | 0.01 |  |  |  |  |
|  | **4 years** |  |  |  |  |  |  |  |  | -0.07 | (-1.99, 1.84) | 0.94 |
|  |  |  |  |  |  |  |  |  |  |  |  |  |

**Footnotes**

Analyses used all available data at each follow-up, with varying numbers for each outcome.

PACE-UP study: N=847-918 at 3 months, 860-931 at 12 months and 639-665 at 3 years. PACE-Lift study: N=258-271 at 3 months, 255-267 at 12 months and 210-220 at 4 years.

All models include treatment group, practice, gender, age at randomisation, and month of baseline accelerometry as fixed effects and household as a random effect in a multi-level linear regression model. The results shown are the change in each Intervention group relative to the change in their Control group.

The effect estimates, 95% confidence intervals and *p*-values were obtained from the model output.

Full references for self-reported pain, HADS depression and anxiety scores, Geriatric Depression Score, FEAR anxiety score, EQ-5D, Exercise Self-Efficacy Score are given in the trial protocols^1,2^

**References**

1. Harris T, Kerry SM, Victor CR, Shah SM, Iliffe S, Ussher M, et al. PACE-UP (Pedometer and consultation evaluation - UP) - a pedometer-based walking intervention with and without practice nurse support in primary care patients aged 45-75 years: study protocol for a randomised controlled trial. Trials. 2013;14:418. doi: 1745-6215-14-418 [pii];10.1186/1745-6215-14-418 [doi].

2. Harris T, Kerry S, Victor C, Ekelund U, Woodcock A, Iliffe S, et al. Randomised controlled trial of a complex intervention by primary care nurses to increase walking in patients aged 60-74 years: protocol of the PACE-Lift (Pedometer Accelerometer Consultation Evaluation - Lift) trial. BMC Public Health. 2013;13. doi: 10.1186/1471-2458-13-5. PubMed PMID: WOS:000313448900001.
