# Supplementary figures and images for: Physical activity levels in adults and older adults 3–4 years after pedometer-based walking interventions: Long-term follow-up of participants from two randomised controlled trials in UK primary care
Source: PLoS Med. 2018 Mar 9;15(3):e1002526. doi: 10.1371/journal.pmed.1002526 (PMC5844512; doi:10.1371/journal.pmed.1002526)

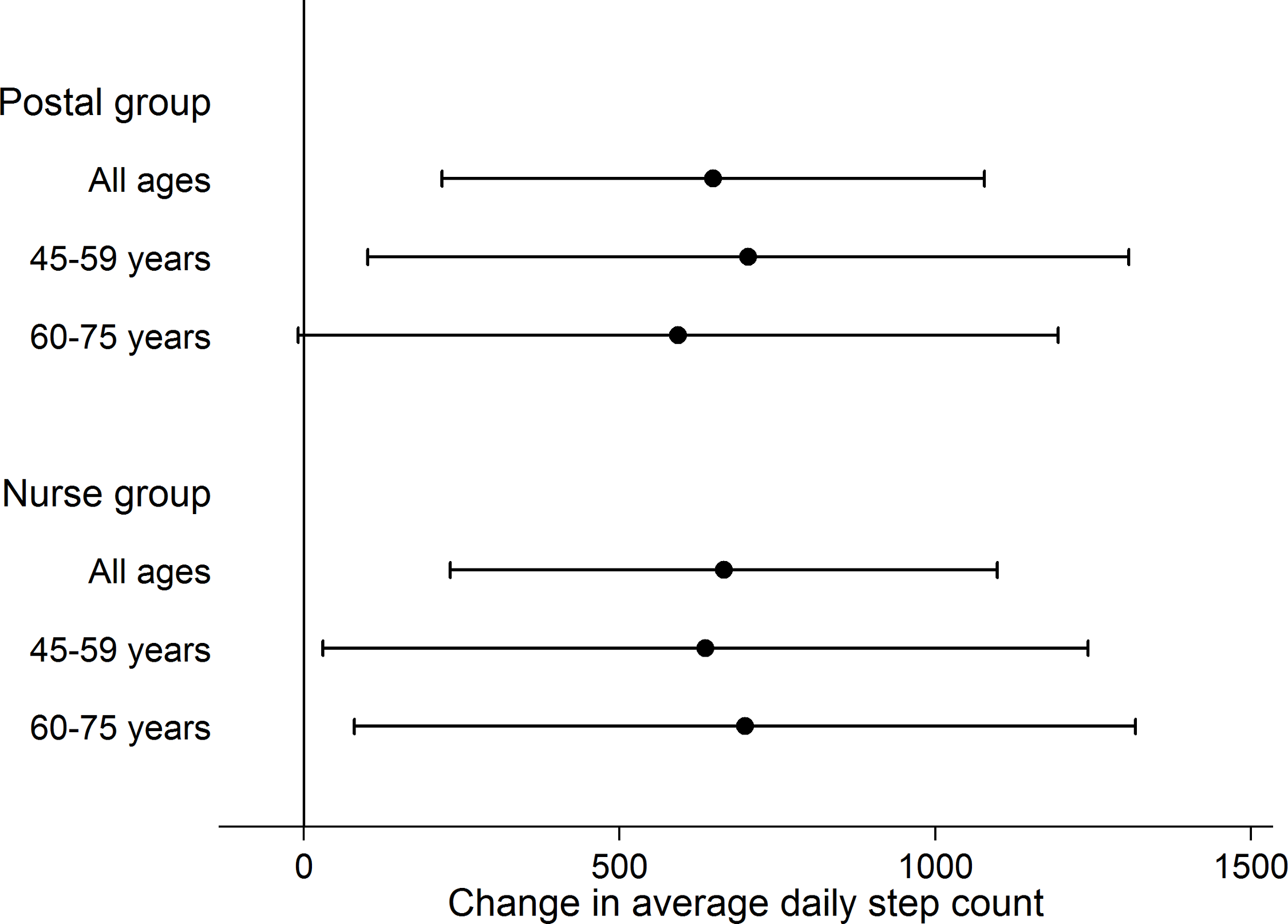

Supplement: S1 Fig — PACE-UP, Pedometer And Consultation Evaluation-UP. (TIF) [file pmed.1002526.s011.tif]

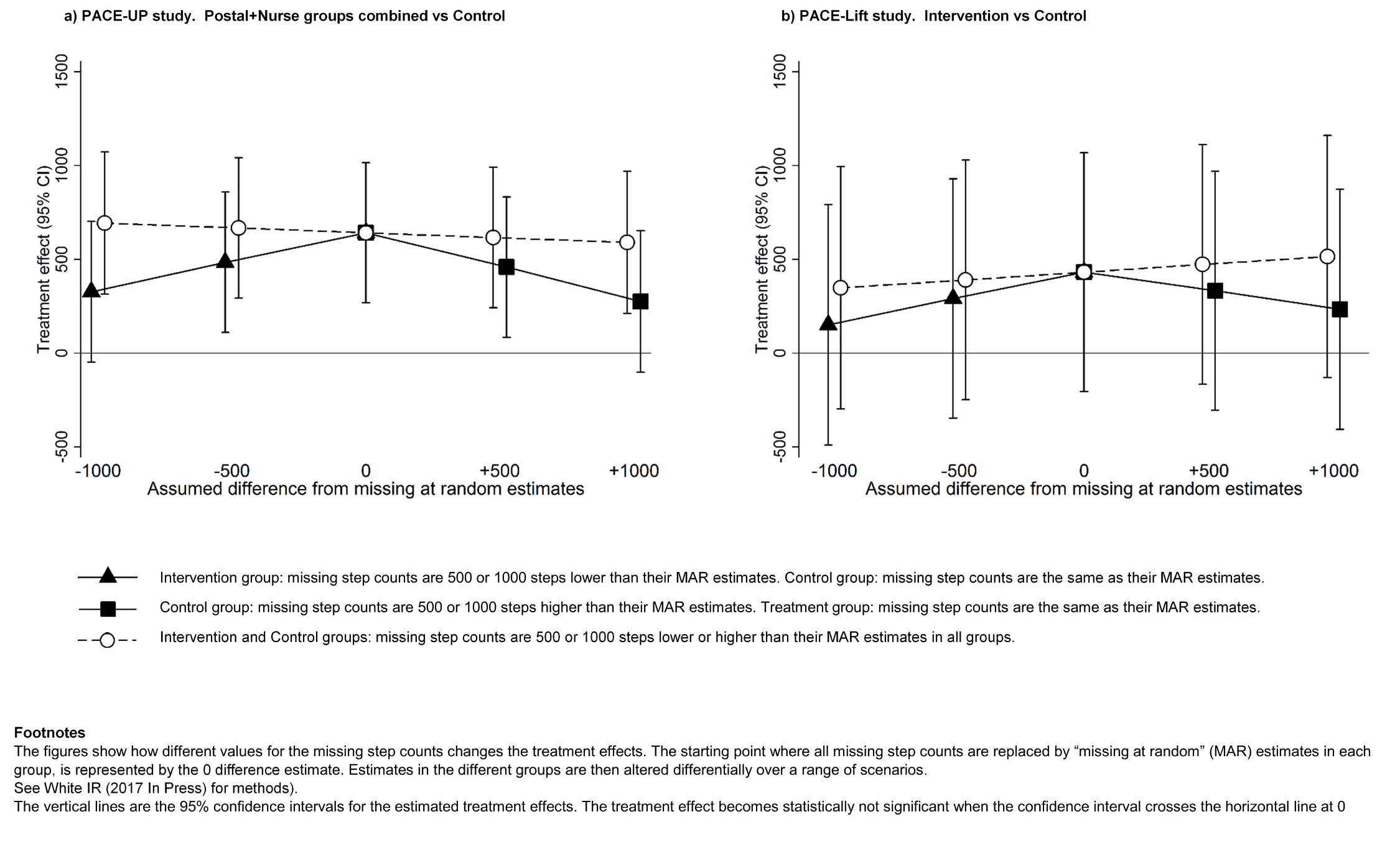

Supplement: S2 Fig — PACE-Lift, Pedometer Accelerometer Consultation Evaluation-Lift; PACE-UP, Pedometer And Consultation Evaluation-UP. (TIF) [file pmed.1002526.s012.tif]
